# Expansion and Diversification of Fluorescent Protein Genes in Fifteen *Acropora* Species during the Evolution of Acroporid Corals

**DOI:** 10.3390/genes12030397

**Published:** 2021-03-11

**Authors:** Rio Kashimoto, Kanako Hisata, Chuya Shinzato, Noriyuki Satoh, Eiichi Shoguchi

**Affiliations:** 1 Marine Genomics Unit, Okinawa Institute of Science and Technology Graduate University, Onna, Okinawa 904-0495, Japan; kanako@oist.jp (K.H.); norisky@oist.jp (N.S.); eiichi@oist.jp (E.S.); 2 Atmosphere and Ocean Research Institute, The University of Tokyo, Kashiwa, Chiba 277-8564, Japan; c.shinzato@aori.u-tokyo.ac.jp

**Keywords:** corals, Acroporidae, *Acropora*, fluorescent proteins, chromoprotein, gene expansion, tandem duplication

## Abstract

In addition to a purple, non-fluorescent chromoprotein (ChrP), fluorescent proteins (FPs) account for the vivid colors of corals, which occur in green (GFP), cyan (CFP), and red (RFP) FPs. To understand the evolution of the coral FP gene family, we examined the genomes of 15 *Acropora* species and three confamilial taxa. This genome-wide survey identified 219 FP genes. Molecular phylogeny revealed that the 15 *Acropora* species each have 9–18 FP genes, whereas the other acroporids examined have only two, suggesting a pronounced expansion of the FP genes in the genus *Acropora*. The data estimates of FP gene duplication suggest that the last common ancestor of the *Acropora* species that survived in the period of high sea surface temperature (Paleogene period) has already gained 16 FP genes. Different evolutionary histories of lineage-specific duplication and loss were discovered among GFP/CFPs, RFPs, and ChrPs. Synteny analysis revealed core GFP/CFP, RFP, and ChrP gene clusters, in which a tandem duplication of the FP genes was evident. The expansion and diversification of *Acropora* FPs may have contributed to the present-day richness of this genus.

## 1. Introduction

Animal color patterns involving fluorescent proteins (FPs) are critical to providing colors to corals and are known to have expanded in these animals, and FPs are perhaps nowhere more exploited than by corals [1,2,3,4]. Coral colors are due primarily to green (GFP), cyan (CFP), and red (RFP) FP emission, as well as in combination with purple or blue non-fluorescent chromoproteins (ChrPs) [5,6,7,8]. Cnidarian GFPs are composed of ~230 amino acids [3,9], which are excited by blue light (maximum 395 nm with a minor peak at 470 nm) and emit green light (peak emission between 509 and 540 nm) [10,11,12]. FPs fluoresce based on the tripeptide chromophore—XYG—[13]. Aequorin is a bioluminescent protein that, through energy transfer, excites GFPs. Aequorin and GFPs were discovered by Osamu Shimomura in the jellyfish *Aequorea victoria* [14]. Since then, FPs have been widely used as reporters of gene expression.

Coral reefs support highly diverse marine species, since up to 25% of known marine species live in coral reefs. However, “coral bleaching”, the collapse of symbiosis between corals and their algal symbionts, occurs frequently in coral reefs throughout tropical oceans, resulting in severe stress for corals. This event is caused by rising seawater temperatures due to climate change [15,16,17], and further catastrophic damage to corals is expected in the future [18]. Stony corals of the genus *Acropora* are some of the most frequent and iconic coral species in tropical oceans. *Acropora* species exhibit various morphologies depending on the clade (arborescent, hispidose, corymbose, and table-shaped) [19]. *Acropora* species are more sensitive to stress than other corals such as the genera *Goniastrea* and *Porites*, making them more susceptible to the effects of bleaching [20]. Since they lost a gene for cystathionine beta-synthase, an enzyme essential for cysteine biosynthesis [21], they depend upon dinoflagellates of the family Symbiodiniaceae and/or other symbiotic organisms to supply this amino acid [22]. On the contrary, the recent decoding of genomes and the subsequent comparative analyses of 18 acroporid corals (the Acroporidae family) shows that *Acropora* diversified 66 Ma ago, at a time when seawater temperatures were much higher than at present [22]. This means the *Acropora* ancestor was resistant to warmer temperatures in the past; however, they might have lost some function regarding their ability to cope with warmer temperatures.

In corals, FPs function as photoprotective host pigments for corals and their symbionts [23]. FPs are excited by high-energy ultraviolet radiation and emit lower-energy visible light. FPs may also reduce oxidative stress in corals, as well as in algal symbionts [24,25,26,27]. Another function of FPs may be to optimize photosynthetic activity in coral polyps [24]. In addition, FPs are likely involved in attracting symbionts to coral hosts [28]. By regulating FP gene expression, corals may emit light at multiple wavelengths to selectively attract different symbiotic algae and prey [28]. Therefore, exploring the number and function of FP candidate genes in corals is important for understanding their survival strategies.

Recently, a number of studies have identified and characterized candidate FP genes in different coral species. Analyses of FP mRNA sequences in *Acropora* species have allowed to categorize FPs based upon whether their emissions occur at short/middle wavelengths (S/MWE) or middle/long wavelengths (M/LWE) [29,30]. The former includes the CFP/GFP group, and the latter contains the GFP/RFP group. Non-fluorescent ChrPs form another group in the FP family [1]. Divergence in the gene copy number between species contributes significantly to genome size and phenotypic variation [31]. CFP genes in *Acropora digitifera* and RFP genes in *Acropora* species may have evolved from gene duplication of paralogous genes [1,32]. One study reported that, in acroporid *Montipora* species, GFPs are translated from different splicing variants [32]. However, we still do not know how many FP genes are present among different acroporids. We expect that the evolution of corals may be more understandable once FP genes have been identified by comparative genomic studies. We recently decoded the genomes of 18 acroporid corals [22]. Therefore, this study was intended to identify genome-wide FP candidate genes in *Acropora* corals.

## 2. Materials and Methods

### 2.1. Genomic Data and Surveys of Fluorescent Protein (FP) Genes

The genomes of the 18 acroporid species, including 15 species of *Acropora* (*Acropora acuminata*,* A. awi*,* A. cytherea*,* A. digitifera*,* A. echinate*,* A. florida*,* A. gemmifera*,* A. hyacinthus*,* A. intermedia*,* A. microphthalma*,* A. muricate*,* A. nasuta*,* A. selago*,* A. tenuis*, and* A. yongei*), *Montipora cactus*, *M. efflorescens*, and *Astreopora myriophthalma*, were sequenced and analyzed by Shinzato et al. [22]. This study was undertaken to shed light on whether *Acropora* corals are genetically equipped to handle warmer oceans.

All samples were collected in Sekisei Lagoon, Okinawa, Japan, and were maintained in aquaria at the Research Center for Subtropical Fisheries, Seikai National Fisheries Research Institute, until spawning [22]. To identify fluorescent protein genes in the assembled genomes, candidate fluorescent gene databases were generated using makeblastdb and a reference fluorescent protein (query) file. BLAST searches (1 × 10^–5^) were performed on protein sequences predicted from genes. The 40 query sequences were selected among well-annotated FPs (Table 1). By surveying Pfam domains (GFP of PF01353), gene models with low similarity to queries were also collected as FP candidates. Predicted proteins with more than one GFP domain (PF01353) were manually separated and were re-annotated with gene ID extensions (_A, _B, or _C). Re-annotated sequences in *Acropora tenuis* were validated by comparing them to transcriptomic data [22,33,34]. A set of 224,869 transcriptome contigs (ID: aten.trinity.all-transcripts.fa) are available on the genome browser of *A. tenuis* [22,34,35].

Additional gene predictions using FGENESH+ [36] were carried out on the incomplete predicted *FP* regions in further analysis. Seventy-seven genes were re-annotated with _FGENESH of the gene ID extension.

### 2.2. Molecular Phylogenetic Analysis

Alignments of FP sequences were performed using MAFFT version 7 [37] and were visualized by Seaview [38]. Maximum likelihood (ML) analyses were executed using Standard RAxML version 8.2.12 [39] with 1000 bootstraps, and trees were drawn with FigTree version 1.4.4 [40] and iTOL v5 [41]. The initial data, which included 306 candidate FPs (Table S1) from 18 acroporid species genomes, were analyzed (Figure S1). The genome of *Stylophora pistillata* (the Pocilloporidae family) [42] was surveyed for FP sequences to supply outgroup sequences for molecular phylogenetic analysis.

**Figure 1:**
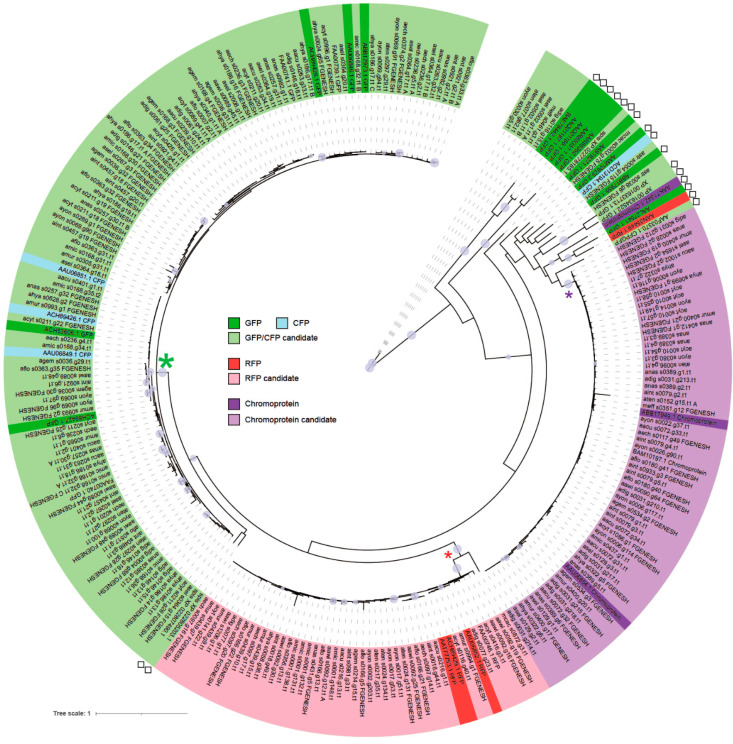
Molecular phylogenetic tree of 255 fluorescent proteins (FPs) from the 18 acroporid species and the other cnidarians. The tree was generated by the maximum likelihood (ML) method. Nodes supported by bootstrap values higher than 70% are shown with a circle using iTOL software. Candidates of the RFPs and chromoproteins in the acroporid corals are classified into two different clades (red and purple stars, respectively). The others are indicated as GFP/CFP candidates that form a large clade (green star) with known GFPs and CFPs. Squares highlights the sequences that are not from acroporid corals.

The preliminary analysis of the aligned sequences from 312 FP candidate genes from 19 coral species included large gaps. Therefore, a more reliable molecular phylogenetic analysis was performed using only 219 FP candidate gene sets from 18 acroporid species, hence suppressing the large gaps (Table S2 and Figure 1).

**Table 1:** Fluorescent proteins used as queries in this study.

Accession Number and Definition in GenBank	Colored Light Emitted in Spectroscopic Analysis
AAU06846.1 green fluorescent protein [*Acropora millepora*]	Green [1]
FAA00739.1 TPA: fluorescent protein 2 [*Acropora digitifera*]	
ABB17973.1 green fluorescent GFP-like protein [*Acropora millepora*]	Green [1]
ACH89428.1 green fluorescent protein FP512 [*Acropora millepora*]	Green [43]
FAA00741.1 TPA: fluorescent protein 4 [*Acropora digitifera*]	
FAA00743.1 TPA: fluorescent protein 6, partial [*Acropora digitifera*]	
FAA00742.1 TPA: fluorescent protein 5, partial [*Acropora digitifera*]	
AAU06851.1 cyan fluorescent protein 2 [*Acropora robusta*]	Cyan [1]
FAA00738.1 TPA: fluorescent protein 1 [*Acropora digitifera*]	
ACH53606.1 green fluorescent-like protein, partial [*Acropora millepora*]	Green [44]
ACH89426.1 cyan fluorescent protein FP484 [*Acropora millepora*]	Cyan [1]
AAU06849.1 cyan fluorescent protein [*Acropora millepora*]	Cyan [1]
ACH89427.1 green fluorescent protein FP497 [*Acropora millepora*]	Green [44]
FAA00740.1 TPA: fluorescent protein 3 [*Acropora digitifera*]	
AAS18271.1 green fluorescent protein 2 [*Astrangia lajollaensis*]	Green [45]
AAT77753.1 colorless GFP-like protein [*Acropora millepora*]	Red [1]
ACH53607.1 red fluorescent-like protein, partial [*Acropora millepora*]	Red [1]
ACH89429.1 red fluorescent protein FP597 [*Acropora millepora*]	Red [1]
AAU06852.1 red fluorescent protein [*Acropora millepora*]	Red [1]
FAA00746.1 TPA: fluorescent protein 10 [*Acropora digitifera*]	
ACD13194.1 green fluorescent GFP-like protein [*Platygyra lamellina*]	Cyan [1]
ABB17955.1 cyan fluorescent GFP-like protein [*Mycedium elephantotus*]	Green [1]
AAM10625.3 green fluorescent protein [*Dendronephthya* sp. *SSAL-2002*]	Green [46]
ABB17949.1 GFP-like chromoprotein [*Goniopora djiboutiensis*]	Non-fluorescent [1]
FAA00744.1 TPA: fluorescent protein 7, partial [*Acropora digitifera*]	
FAA00745.1 TPA: fluorescent protein 9, partial [*Acropora digitifera*]	
BAM10197.1 fluorescent protein 8 [*Acropora digitifera*]	
AAU06854.1 chromoprotein [*Acropora millepora*]	Non-fluorescent [1]
AAG16224.1 red fluorescent protein [*Discosoma* sp. *SSAL-2000*]	Red [47]
AAF03370.1 fluorescent protein FP483 [*Discosoma striata*]	
XP_001634522.1 predicted protein [*Nematostella vectensis*]	
XP_001633713.1 predicted protein [*Nematostella vectensis*]	
AAN05449.1 red fluorescent protein FP611 [*Entacmaea quadricolor*]	Red [48]
AAL27541.1 GFP-like chromoprotein [*Condylactis passiflora*]	Green [49]
AAK71342.1 cgigFP-g [*Condylactis gigantea*]	Non-fluorescent [46]
AAQ01187.1 green fluorescent protein 2 [*Pontella meadi*]	Green [50]
AAQ01186.1 green fluorescent protein 1 [*Pontella meadi*]	Green [50]
BAE78442.1 green fluorescent protein [*Chiridius poppei*]	Green [51]
AAR85351.1 green fluorescent protein 2 [*Anthomedusae* sp. *SL-2003*]	Green [50]
AAR85350.1 green fluorescent protein 1 [*Anthomedusae* sp. *SL-2003*]	Green [50]

### 2.3. Classification into GFP/CFP, RFP, and ChrP Groups and Chromophore Sequences

In the preliminary analysis (Figure S1), we used 306 candidate FPs to make a phylogenetic tree, with the fluorescent proteins used as queries in this study for the identification of predicted color light (Figure S1). The RFPs and ChrP clades were supported by high bootstrap (≥90%), while the others were classified into a GFP/CFP group.

Sequences of GFPs, CFPs, RFPs, and ChrPs of confirmed colored light emitted in spectroscopic analysis (Table 1) were used for reliable molecular phylogenetic analysis of the candidates of 219 complete FPs (Figure 1). The amino acid sequences of GFPs/CFPs, RFPs, and ChrPs were aligned, and the major chromophore sequences (GFPs/CFPs and ChrPs: QYG; RFP: DYG) were checked [30].

### 2.4. Gene Duplication and Gene Loss Analysis

The Notung software [52] was used to estimate gene duplications and losses [53]. The species tree containing 19 coral species based on the protein sequences was used as the input file [22,42].

For the gene tree, in the preliminary analysis, the data of 312 FPs from 19 species of corals that included partial sequences were used and the total FPs, GFPs/CFPs, RFPs and chromoprotein number of duplication and losses were counted (Figure S2).

**Figure 2:**
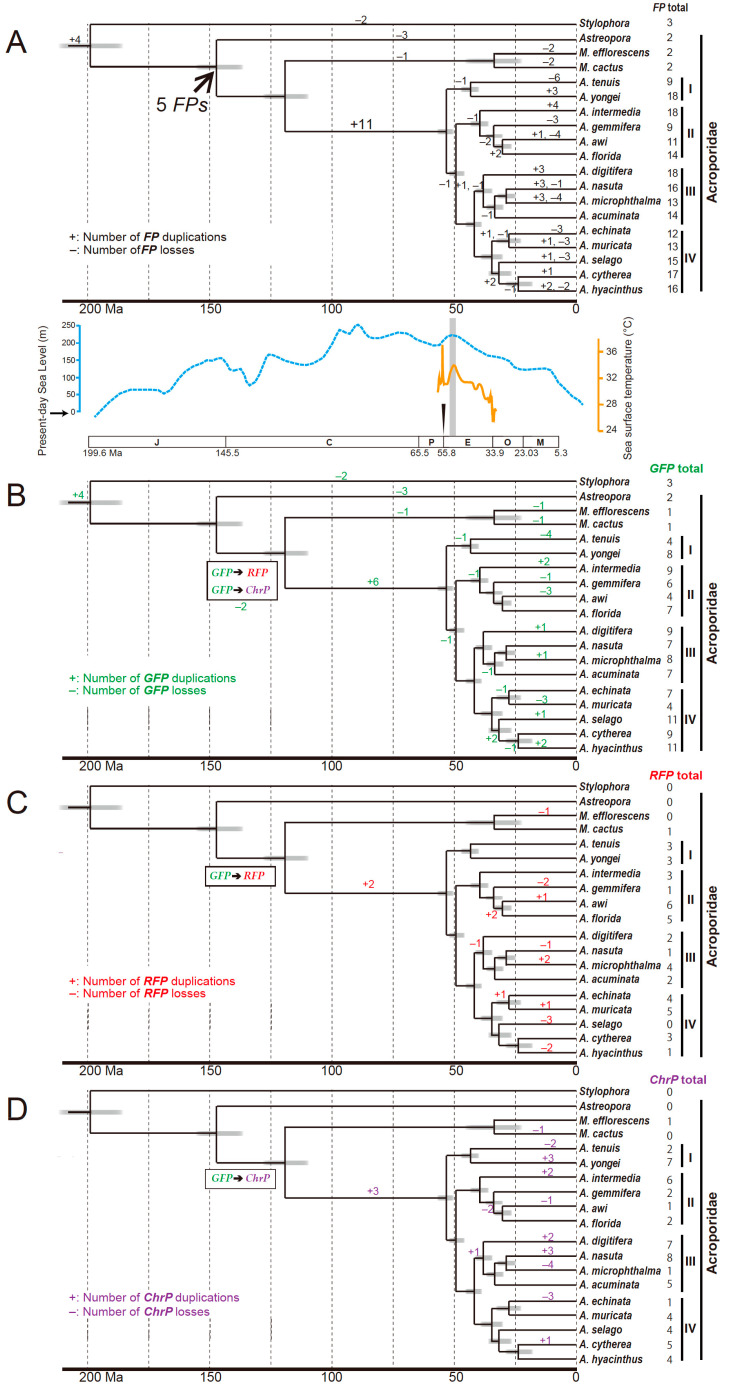
Expansions of the fluorescent protein (FP) genes of the genus *Acropora* with estimated divergence times of the acroporid corals indicated at the bottom of each tree. Numbers of genes duplicated (+) or lost (–) are indicated at each branch. (**A**) FP gene duplications and losses. It is inferred that the Acroporidae ancestor had five *FPs*. Species trees with divergence time estimates, changes in sea level, and sea surface temperature are indicated [22]. Expected sea level changes are shown with a blue dotted line [59]. Estimated tropical sea surface temperature of the Eocene is indicated with an orange line [60]. The Paleocene–Eocene thermal maximum (PETM) is indicated with an arrowhead, and the Early Eocene climatic optimum (EECO) is highlighted in light grey. An approximate geological time scale is shown at the bottom. J, Jurassic period; C, Cretaceous period; P, Paleocene; E, Eocene; O, Oligocene; M, Miocene. The history of *FP* duplications and losses are classified into those of *GFP*/*CFPs* (**B**), *RFPs* (**C**), and *ChrPs* (**D**). Squares show that putative *GFP* evolved into *RFP* or *ChrP*.

For reliable data analysis, the candidate of 219 FPs from 18 species of corals that included complete sequence were used and the total FPs, GFPs/CFPs, RFPs and chromoprotein number of duplication and losses were counted (Figure 2 and Table S3).

### 2.5. Synteny Analysis

Using the genome browser for eight species of *Acropora* [54], gene organization was checked [35]. Gene annotations on the browsers were performed with Blast2GO, Pfam domain searches, and InterProScan 5.25–64.0 [55], or with BLAST searches of the NCBI reference sequences [56]. The syntenic regions were also analyzed and visualized using zPicture software [57]. The genomic sequence of every two species that encoded FP genes in the scaffolds was uploaded on the website [58]. The sequences were aligned on the BLASTZ program with the default setting.

## 3. Results

### 3.1. Fluorescent Protein Genes in 18 Coral Species

We identified candidate FP genes by analyzing the assembled genomes of 18 acroporid species (*Acropora* and three other confamilial taxa) using BLASTP searches with 40 protein sequences as queries (Table 1) and using Pfam domain searches. Two hundred and ninety-six predicted proteins were selected as candidates. Some longer sequences with two or three GFP domains (PF01353) likely represent errors of in silico gene prediction, as was confirmed when they were compared to query data (Table 1). Longer regions in these proteins were manually deleted. Transcriptome data of *A. tenuis* [22,33,34] supported this split. We added _A, _B, or _C to the gene model IDs to indicate these splits (Figure S2 and Table S1). As a result, we found 306 FP candidates in the 18 acroporid genomes. Additionally, these alignments detected ~160 incomplete sequences with large gap regions. Gene predictions on these incomplete predicted FP regions from the initial analysis were re-analyzed by FGENESH+ [36]. Following this, 77 genes were re-annotated (Table S2).

To perform reliable analysis, we defined the complete sequences by reference to full-length sequences (Table 1) and three FP proteins (XP_022805203.1, XP _022807490.1.1, and XP_022778185.1) of the coral *Stylophora pistillata* (Figure S3). Incomplete sequences with large gaps were removed. Finally, 219 FP sequences from 306 candidates were used for further analysis (Table S2). We checked whether these acroporid FPs have a tripeptide chromophore motif XYG, in which -X is variable but most often Q [13,30]. Only six of the 219 predicted proteins showed divergent tripeptide in which XYG is replaced by XFG. By comparing the gene numbers encoding FPs between species, we found that the 15 *Acropora* species have 9–18 FP genes, whereas the two *Montipora* species and *Astrepora myriophthalma* have only two (Table 2). The 15 *Acropora* species were classified into four clades (I, II, III, and IV) by molecular phylogenetic analysis [22] (Figure 2). The average number of FP genes for each clade was around 14, suggesting that the number of FP genes heavily changes from the outgroup to the *Acropora* species.

### 3.2. Emission Color Prediction of Fluorescent Proteins by Molecular Phylogenetic Analysis

Using molecular phylogenetic analysis, we tried to predict the emission colors of the 219 FPs present in the 18 acroporid species (Table 2 and Table S2). The emissions of 27 FPs have previously been reported by spectroscopic analysis (Table 1). By comparing those spectroscopically analyzed 25 FPs (except for ACH53607.1 red fluorescent-like protein, partial [*Acropora millepora*] and AAG16224.1 red fluorescent protein [*Discosoma* sp. SSAL-2000]) to the FPs of each acroporid genome, we predicted the color emissions of the 219 FP candidate genes. In our molecular phylogenetic analysis, the RFPs and ChrP clades were relatively well supported with high bootstrap values (two small asterisks in Figure 1). The GFPs/CFPs of the acroporid corals formed a clade with a high bootstrap value (large asterisk in Figure 1). However, there were low bootstrap values allowing to separate GFPs and CFPs. Therefore, these GFPs and CFPs were grouped into the GFP/CFP category. Our analysis results show that the GFP/CFP clade is predominant, consistent with previous studies in which GFP expression was best confirmed in *Acropora* species [1].

### 3.3. Expansion of the Number of Fluorescent Protein Genes in Acropora

To determine whether the FP gene is duplicated frequently within *Acropora*, we examined the FP gene duplication and loss event using Notung software (Table S3 and Figure S4) [53]. Our analysis predicted that the ancestor of the Acroporidae family originally had five FP genes (Figure 2A). We confirmed that 11 genes were duplicated after the *Montipora* lineage’s divergence and presumably constituted the ancestral complement of the four clades of *Acropora* (Figure 2A). This step likely corresponds to the Cretaceous and Paleocene periods (see the geological time scale indicated at the bottom of Figure 2A), predicted as the periods of high sea level (blue dotted line in Figure 2A). These 11 gene duplication events suggest that the FP gene expanded only in the common lineage of the 15 *Acropora* species. In contrast, in the genera of *Astreopora* and *Montipora,* gene losses are predicted to have occurred. Lineage-specific duplications and losses of FP genes have occurred continuously in the 15 species of *Acropora* after the warmer period (Figure 2A). The period of a small number of gene losses and duplications indicates Eocene, in which the sea surface temperature dropped (orange line in Figure 2A). *FP* duplications and losses have occurred four times in total in each of the lineages, from the *Acropora* ancestor to *A. hyacinthus*, suggesting a constant refinement in FP function diversification.

According to our *FP* classification (Figure 1), the highest number of GFP/CFP gene duplications occured in Clade IV (Figure 2B). On the contrary, *GFP/CFP* duplication was not predicted in the Clade I lineage. Interestingly, we observed a loss of GFP/CFP genes in each of the four clades. It has been assumed that *GFPs* have evolved repeatedly and independently into *RFP* and *ChrPs* [1,30]. We hypothesized that the first *RFP* and *ChrP* emerged in the common ancestor of *Montipora* and *Acropora* species (see also in squares of Figure 2B–D). Further *RFP* duplications probably also happened in *A. awi* and *A. florida* in Clade II after they diverged from *A. gemmifera* (Figure 2C). Meanwhile, from our analysis, we could not confirm RFP duplications and losses within Clade I. Lineage-specific duplications of *ChrPs* were predicted mainly in Clade III (Figure 2D), roughly corresponding to the Oligocene and Miocene periods. The genomes of *A. muricata*, *A. selago*, and *A. hyacinthus* from Clade IV seem to have retained the four *ChrPs* of the *Acropora* ancestor.

### 3.4. Localization of Fluorescent Protein Genes in Acropora Genomes

**Figure 3:**
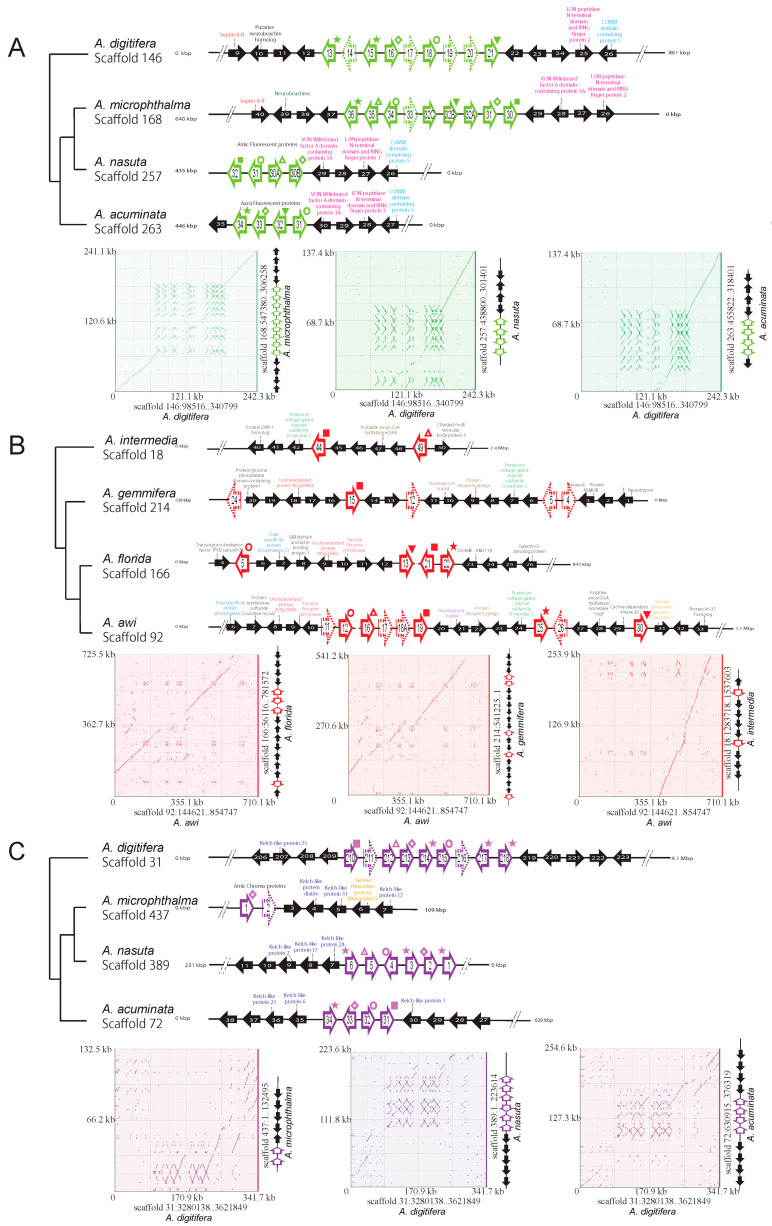
Three core gene clusters, including nine FP genes and syntenic regions in *Acropora* genomes. Synteny analysis showed the probability of extensive tandem gene duplications or losses at three genomic locations in the *Acropora* lineages. (**A**) *GFP/CFP* (green arrow) gene clusters in Clade III *Acropora* (*A. digitifera,* *A. nasuta,* *A. microphthalma,* and *A. acuminata*). (**B**) *RFP* (red arrow) clusters in Clade II (*A. intermedia,* *A. gemmifera,* *A. awi,* and *A. florida*). (**C**) *ChrP* (purple arrow) clusters in Clade III (*A. digitifera,* *A. nasuta,* *A. microphthalma,* and *A. acuminata*). The clades correspond to Figure 2. The orthologous relationships between species are shown as symbols (★, ○, ■, ◇, and △) that correspond to Figure S5. The standard arrow indicates predicted complete FP genes (Table S2), and the dashed arrow indicates the predicted incomplete FP genes from this study (Table S1). Black arrows indicate neighboring genes to *FPs*.

In light of our genomic surveys (Table 2 and Figure 1), we further examined whether tandem or other types of gene duplications occurred among the FP genes of the coral species. By comparing the genomic regions, we specifically examined the FP gene orthology and searched for FP gene neighbors, in addition to comparing the genomic locations of *FPs* within clades. Orthologous relationships were estimated by molecular phylogeny (Figure S5). We found that the largest number of FP genes were present in a cluster on Scaffold 146 of *A. digitifera*, Scaffold 168 of *A. microphthalma*, Scaffold 92 of *A. awi*, and Scaffold 31 of *A. digitifera* (Figure 3 and Table S1), including nine FP genes in the preliminary analysis. Then, we selected other species scaffolds for gene localization analysis (Figure 3 and Table S1), in which the largest number of FP genes in each of Clades I–IV was included. We expected this largest number of FP genes in a cluster to reveal orthologous and syntenic relationships between the different genes examined in these species (Figure 3).

The molecular phylogenetic tree, including 222 predicted proteins, showed clear orthologous relationships between *A. digitifera GFPs/CFPs* in the gene cluster and other *GFPs/CFPs* of Clade III, supporting the presence of a core gene cluster in the Clade III ancestor (Figure 3A and Figure S5). In GFP/CFP genes of Clade III *Acropora*, the orthologous relationships among *GFPs/CFPs* indicated that two *GFPs/CFPs* (adig_s0146.g13 and adig_s0146.g15) of *A. digitifera* occurred via tandem duplication (Figure 3A). The *GFP* orthologous relationships suggested that similar gene re-arrangements in *GFP/CFP* clusters occurred repeatedly. Neighboring genes were conserved among the *GFP/CFP* cluster of Clade III *Acropora*. When dot-plots visualized synteny region among the four genomes of Clade III of the *Acropora* species, opposite gene directions of GFPs/CFPs and chromoproteins were found (lower part of Figure 3A), suggesting that an inversion event of GFP/CFP and chromoprotein clusters may have happened in the Clade III lineage. On the contrary, the order and directions of neighboring genes were likely to be more conserved than those of *GFPs*.

The RFP genes of Clade II *Acopora* were duplicated more than those of the others. Analysis of the orthologous relationship revealed two duplicated RFP genes in the ancestor of *A. florida* and *A. awi* on Scaffolds 166 and 92, respectively (Figure 3B). Seven genes for the other protein families were present between the duplicated *RFPs* (aflo_s0166.g5 and aflo_s0166.g13) of *A. floridae* (Figure 3B). The dot-plot analysis supported that the synteny was conserved among the RFP genes contained in this genomic location, suggesting a translocation event soon after tandem duplication or, alternatively, another duplication mechanism.

The *A. digitifera* genome also had a large gene cluster for putative ChrPs in addition to the *GFP/CFP* cluster (Figure 3C). The orthologous relationships among the *ChrPs* of Clade III showed that lineage-specific gene duplication in *A. digitifera* and *A. nasuta* occurred twice independently on the gene cluster (see genes with stars in Figure 3C and Figure S5). Synteny analysis suggests that the neighboring genes were less conserved than those of the *GFP/CFP* cluster. In summary, our study indicates that the gene clusters for GFP/CFP and ChrP were formed separately in the *Acropora* ancestor and experienced lineage-specific tandem duplications. The *ChrP* cluster of Clade III *Acropora* included genes for a kelch-repeat superfamily that is involved in protein–protein interactions [61]. This family is conserved in diverse organisms spanning from viruses, plants, and fungi to mammals [61], suggesting that the *ChrP* cluster of the *Acropora* ancestor may have been conserved with those neighboring genes.

## 4. Discussion

In this paper, we identified 219 FP candidate genes from 18 acroporid genomes that encode proteins with potentially complete amino acid sequences. Our results show the existence of 9–18 FP genes in the 15 *Acropora* species, but only two FP genes in other analyzed acroporid species (Table 2). Our preliminary analysis therefore implies that 306 *FPs* might be encoded in the 18 acropoid genomes. However, because draft genomes include gaps, some predicted FP genes seem to encode only partial GFP domains. Improving the *Acropora* genome assemblies will certainly allow to identify additional *FPs*. To check computational gene predictions, we used the transcriptomic data of *A. tenuis* [22]. In a previous study, the expressions of *A. tenuis* FP genes were reported, and the differential expressions of *A. tenuis FPs* were examined [33]. One of the GFP genes, aten_s0077.g62, had weak expression [33] and might belong to orphan genes in the *Acropora* lineage (see Figure 1). Since these orphan genes are considered necessary taxon-specific developmental adaptations that play a role in interactions with the environment [62], we plan to analyze carefully in the future the expression and function of these genes.

*Acropora* species produce various colors through FP emission, which may contribute to their richness [24]. Indeed, previous reports have suggested that bleaching events trigger colorization in reef-building corals [23,33]. In previous reports, 10 FP genes were estimated from the first draft genome of *A. digitifera* [5]. In this new study, we identified 18 FP genes from *A. digitifera* using a new version of the genome. This new large number of candidate FPs was also confirmed by transcriptome analysis of FP candidates [29]. At the beginning of our research, we attempted to separate the FPs into two groups, namely, GFPs and CFPs. However, as we used only genome and transcriptome data for this color classification, we faced many difficulties, since, as previously mentioned [6], the nucleotide sequence only was insufficient to identify the emission color between CFPs and GFPs. This color assignation should be confirmed by adding CFP emission color data to reference nucleotide data. Although, we grouped FPs into GFPs/CFPs, RFPs, and ChrPs by molecular phylogenetic analysis, it may be possible using the newly analyzed data to predict the emissions of all FPs by analyzing the chromophore (XYG) region. Indeed, our predictions for 219 FP proteins suggest that most chromophores in GFPs/CFPs and ChrPs are QYG, whereas those in RFPs are DYG (Figure S3), as mentioned in a previous study [29]. Minor chromophores (EYG, HYG, or MYG) were associated with GFP/CFP candidates, whereas TYG was associated with RFP candidates in our analyzed data (Figure S3) [1,29,33,43,44,45,46,47,48,49,50,51].

**Figure 4:**
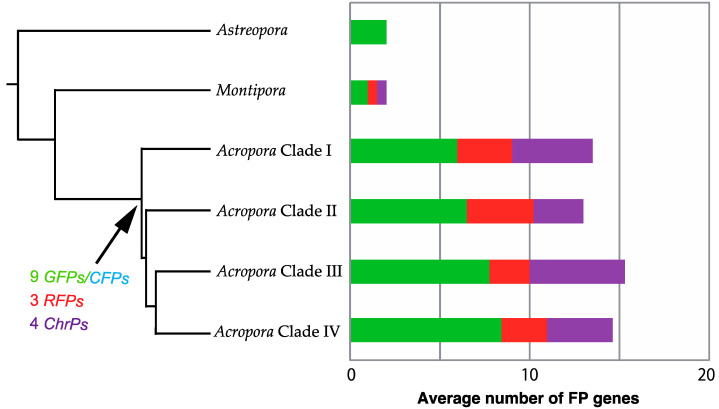
Estimated numbers of fluorescent genes in the *Acropora* ancestor and diversification in the descendant lineages. It is hypothesized that ~16 FP genes have been conserved in *Acropora*. RFP and GFP/CFP genes may have diversified more in Clades II and IV, respectively.

We documented a large *FP* expansion among the 15 species of *Acropora* by comparing them to three other acroporid taxa (Figure 1). Our analysis estimated that nine *GFP/CFPs*, three *RFPs*, and four *ChrPs* were present in the common ancestor of *Acropora* Clades I–IV after divergence from the *Montipora* lineage (Figure 4). Data estimation of the gene duplications and losses suggested that the gene expansion of *Acropora FPs* occurred in the early lineage, corresponding to a period of high sea level (Figure 2). FP losses occurred in each lineage during the diversification to *Acropora* Clades I–IV, and the species-specific gene duplications and losses occurred repeatedly in the Oligocene period. Those repeated *FP* duplications and losses may have contributed to the increase in the richness of *Acropora*.

Genomic localization of the FP genes in *Acropora* suggested that *GFPs/CFPs*, *RFPs*, and *ChrPs* were tandemly duplicated in three major regions (Figure 3). Some tandemly duplicated *GFPs/CFPs*, *RFPs*, and *ChrPs* had orthologous relationships with genes in a cluster with other species of *Acropora* (Figure 3 and Figure S5). Previous reports have suggested that FP emissions increase when corals adapt to environmental change [23,63]. Before ~66 Ma ago, the early ancestor of *Acropora* with tandemly duplicated FPs may have survived a warmer period with increased fluorescent lights and then diversified soon after during the Ice Age (Figure 2A). The expansion of *FPs* in three chromosomal regions of the early ancestor of *Acropora* may have allowed diversification into four clades and may have contributed to the environmental adaptation and speciation of these fascinating corals.

## 5. Conclusions

The analysis of fluorescent protein candidates from 18 species of acroporid corals revealed a dramatic difference in the number of fluorescent proteins between the genus *Acropora* and three confamilial taxa. This suggests that the expansion and diversification of fluorescent protein genes occurred during the evolution of reef-building corals before the appearance of the common ancestor of the extant *Acropora* species (Figure 3). It is likely that the ancestral fluorescent protein gene was expanded by tandem duplication, and that orthologous genes were conserved among *Acropora* species. It is possible that other cnidarian species might acclimatize to climate change by modifying their FP emission colors. Our findings can therefore be potentially applied to regenerating reef-building corals resistant to global warming.

## Figures and Tables

**Table 2:** The number of complete fluorescent protein (FP) candidate genes in acroporid coral genomes.

Coral Species	*GFP/CFP*	*RFP*	*ChrP*	FP Total
*Astrepora myriophthalama*	2	0	0	2
*Montipora efflorescens*	1	0	1	2
*Montipora cactus*	1	1	0	2
*A. tenuis* (I) ^1^	4	3	2	9
*A. yongei* (I)	8	3	7	18
*A. intermedia* (II)	9	3	6	18
*A. gemmifera* (II)	6	1	2	9
*A. awi* (II)	4	6	1	11
*A. florida* (II)	7	5	2	14
*A. digitifera* (III)	9	2	7	18
*A. nasuta* (III)	7	1	8	16
*A. microphthalma* (III)	8	4	1	13
*A. acuminata* (III)	7	2	5	14
*A. echinata* (IV)	7	4	1	12
*A. muricata* (IV)	4	5	4	13
*A. selago* (IV)	11	0	4	15
*A. cytherea* (IV)	9	3	5	17
*A. hyacinthus* (IV)	11	1	4	16

^1^ Parentheses show *Acropora* clades [22]. GFP, green FP; CFP, cyan FP; RFP, red FP; ChrP, chromoprotein.

## Data Availability

A set of 224,869 transcriptome contigs (ID: aten.trinity.all-transcripts.fa) are available online at https://marinegenomics.oist.jp/aten/viewer/download?project_id=97 (accessed on 8 March 2021).

## Supplementary Materials

The following are available online at https://www.mdpi.com/2073-4425/12/3/397/s1: Figure S1. Fluorescent protein (FP) molecular phylogeny of 15 species of *Acropora*, two species of *Montipora*, and one species of *Astreopora* in the family Acroporidae; Figure S2. Expansions of the fluorescent protein (FP) genes of the genus *Acropora* with estimated divergence times of acroporid corals by preliminary analysis; Figure S3. Alignments of the predicted FP proteins of 18 acroporid corals; Figure S4. A phylogenetic tree showing the node IDs from the Notung output; Figure S5. Ortholog prediction by the molecular phylogenetic tree of the Acroporidae FPs from Notung software; Table S1. FP genes for preliminary analysis; Table S2. Analyzed FP genes; Table S3. Analyzed FP duplications and losses.

## References

[1] Alieva N.O., Konzen K.A., Field S.F., Meleshkevitch E.A., Hunt M.E., Beltran-Ramirez V., Miller D.J., Wiedenmann J., Salih A., Matz M.V. (2008). Diversity and evolution of coral fluorescent proteins. PLoS ONE.

[2] Dove S.G., Hoegh-Guldberg O., Ranganathan S. (2001). Major colour patterns of reef-building corals are due to a family of GFP-like proteins. Coral Reefs.

[3] Mazel C.H., Lesser M.P., Gorbunov M.Y., Barry T.M., Farrell J.H., Wyman K.D., Falkowski P.G. (2003). Green-fluorescent proteins in Caribbean corals. Limnol. Oceanogr..

[4] Matz M.V., Marshall N.J., Vorobyev M. (2006). Are Corals Colorful?. Photochem. Photobiol..

[5] Shinzato C., Shoguchi E., Tanaka M., Satoh N. (2012). Fluorescent protein candidate genes in the coral *Acropora digitifera* genome. Zool. Sci..

[6] Matz M.V., Fradkov A.F., Labas Y.A., Savitsky A.P., Zaraisky A.G., Markelov M.L., Lukyanov S.A. (1999). Fluorescent proteins from nonbioluminescent Anthozoa species. Nat. Biotechnol..

[7] Kelmanson I.V., Matz M.V. (2003). Molecular basis and evolutionary origins of color diversity in great star coral montastraea cavernosa (Scleractinia: Faviida). Mol. Biol. Evol..

[8] Field S.F., Bulina M.Y., Kelmanson I.V., Bielawski J.P., Matz M.V. (2006). Adaptive evolution of multicolored fluorescent proteins in reef-building corals. J. Mol. Evol..

[9] Prasher D.C., Eckenrode V.K., Ward W.W., Prendergast F.G., Cormier M.J. (1992). Primary structure of the *Aequorea victoria* green-fluorescent protein. Gene.

[10] Mazel C.H. (1997). Coral fluorescence characteristics: Excitation/emmission spectra, fluorescence efficiences, and ontributi to apparent reflectance. Proceedings of the one Ocean Optics XIII.

[11] Morin J.G., Hastings J.W. (1971). Energy transfer in a bioluminescent system. J. Cell Physiol..

[12] Ward W.W., Cody C.W., Hart R.C., Cormier M.J. (1980). Spectrophotometric identity of the energy transfer chromophores in renilla and aequorea green-fluorescent proteins. Photochem. Photobiol..

[13] Henderson J.N., Remington S.J. (2005). Crystal structures and mutational analysis of amFP486, a cyan fluorescent protein from Anemonia majano. Proc. Natl. Acad. Sci. USA.

[14] Shimomura O., Johnson F.H., Saiga Y. (1962). Extraction, purification and properties of aequorin, a bioluminescent protein from the luminous hydromedusan, Aequorea. J. Cell Comp. Physiol..

[15] Hughes T.P., Kerry J.T., Álvarez-Noriega M., Álvarez-Romero J.G., Anderson K.D., Baird A.H., Babcock R.C., Beger M., Bellwood D.R., Berkelmans R. (2017). Global warming and recurrent mass bleaching of corals. Nature.

[16] Nakamura T. (2017). Mass coral bleaching event in Sekisei lagoon observed in the summer of 2016. J. Jpn. Coral Reef Soc..

[17] Head C.E.I., Bayley D.T.I., Rowlands G., Roche R.C., Tickler D.M., Rogers A.D., Koldewey H., Turner J.R., Andradi-Brown D.A. (2019). Coral bleaching impacts from back-to-back 2015–2016 thermal anomalies in the remote central Indian Ocean. Coral Reefs.

[18] Perry C.T., Alvarez-Filip L., Graham N.A.J., Mumby P.J., Wilson S.K., Kench P.S., Manzello D.P., Morgan K.M., Slangen A.B.A., Thomson D.P. (2018). Loss of coral reef growth capacity to track future increases in sea level. Nature.

[19] Wallace C.C. (1999). Staghorn Corals of the World: A Revision of the Coral Genus Acropora (Scleractinia; Astrocoeniina; Acroporidae) Worldwide, with Emphasis on Morphology, Phylogeny and Biogeography.

[20] Loya Y., Sakai K., Yamazato K., Nakano Y., Sambali H., Woesik R. (2001). van Coral bleaching: The winners and the losers. Ecol. Lett..

[21] Shinzato C., Shoguchi E., Kawashima T., Hamada M., Hisata K., Tanaka M., Fujie M., Fujiwara M., Koyanagi R., Ikuta T. (2011). Using the *Acropora digitifera* genome to understand coral responses to environmental change. Nature.

[22] Shinzato C., Khalturin K., Inoue J., Zayasu Y., Kanda M., Kawamitsu M., Yoshioka Y., Yamashita H., Suzuki G., Satoh N. (2020). Eighteen coral genomes reveal the evolutionary origin of *Acropora* strategies to accommodate environmental changes. Mol. Biol. Evol..

[23] Bollati E., D’Angelo C., Alderdice R., Pratchett M., Ziegler M., Wiedenmann J. (2020). Optical Feedback Loop Involving Dinoflagellate Symbiont and Scleractinian Host Drives Colorful Coral Bleaching. Curr. Biol..

[24] Salih A., Larkum A., Cox G., Kühl M., Hoegh-Guldberg O. (2000). Fluorescent pigments in corals are photoprotective. Nature.

[25] Palmer C.V., Modi C.K., Mydlarz L.D. (2009). Coral fluorescent proteins as antioxidants. PLoS ONE.

[26] Roth M.S. (2014). The engine of the reef: Photobiology of the coral–algal symbiosis. Front. Microbiol..

[27] Bou-Abdallah F., Chasteen N.D., Lesser M.P. (2006). Quenching of Superoxide Radicals by Green Fluorescent Protein. Biochim. Biophys. Acta.

[28] Aihara Y., Maruyama S., Baird A.H., Iguchi A., Takahashi S., Minagawa J. (2019). Green fluorescence from cnidarian hosts attracts symbiotic algae. Proc. Natl. Acad. Sci. USA.

[29] Takahashi-Kariyazono S., Sakai K., Terai Y. (2018). Presence–absence polymorphisms of highly expressed fp sequences contribute to fluorescent polymorphisms in acropora digitifera. Genome Biol. Evol..

[30] Takahashi-Kariyazono S., Gojobori J., Satta Y., Sakai K., Terai Y. (2016). Acropora digitifera encodes the largest known family of fluorescent proteins that has persisted during the evolution of *Acropora* species. Genome Biol. Evol..

[31] Suryawanshi V., Talke I.N., Weber M., Eils R., Brors B., Clemens S., Krämer U. (2016). Between-species differences in gene copy number are enriched among functions critical for adaptive evolution in arabidopsis halleri. BMC Genom..

[32] Takahashi-Kariyazono S., Satta Y., Terai Y. (2015). Genetic diversity of fluorescent protein genes generated by gene duplication and alternative splicing in reef-building corals. Zool. Lett..

[33] Satoh N., Kinjo K., Shintaku K., Kezuka D., Ishimori H., Yokokura A., Hagiwara K., Hisata K., Kawamitsu M., Koizumi K. (2021). Color morphs of the coral, Acropora tenuis, show different responses to environmental stress and different expression profiles of fluorescent-protein genes. G3 Genes|Genomes|Genetics.

[34] Koyanagi R., Takeuchi T., Hisata K., Gyoja F., Shoguchi E., Satoh N., Kawashima T. (2013). MarinegenomicsDB: An integrated genome viewer for community-based annotation of genomes. Zool. Sci..

[35] OIST Marine Genomics Unit Genome Browser. https://marinegenomics.oist.jp/aten/viewer/download?project_id=97.

[36] Solovyev V., Kosarev P., Seledsov I., Vorobyev D. (2006). Automatic annotation of eukaryotic genes, pseudogenes and promoters. Genome Biol..

[37] Katoh K., Standley D.M. (2013). MAFFT multiple sequence alignment software version 7: Improvements in performance and usability. Mol. Biol. Evol..

[38] Gouy M., Guindon S., Gascuel O. (2010). Seaview version 4: A multiplatform graphical user interface for sequence alignment and phylogenetic tree building. Mol. Biol. Evol..

[39] Stamatakis A. (2014). RAxML version 8: A tool for phylogenetic analysis and post-analysis of large phylogenies. Bioinformatics.

[40] FigTree. http://tree.bio.ed.ac.uk/software/figtree/.

[41] Ciccarelli F.D., Doerks T., von Mering C., Creevey C.J., Snel B., Bork P. (2006). Toward automatic reconstruction of a highly resolved tree of life. Science.

[42] Voolstra C.R., Li Y., Liew Y.J., Baumgarten S., Zoccola D., Flot J.-F., Tambutté S., Allemand D., Aranda M. (2017). Comparative analysis of the genomes of *Stylophora pistillata* and *Acropora digitifera* provides evidence for extensive differences between species of corals. Sci. Rep..

[43] D’Angelo C., Denzel A., Vogt A., Matz M.V., Oswald F., Salih A., Nienhaus G.U., Wiedenmann J. (2008). Blue light regulation of host pigment in reef-building corals. Mar. Ecol. Prog. Ser..

[44] Smith-Keune C., Dove S. (2008). Gene expression of a green fluorescent protein homolog as a host-specific biomarker of heat stress within a reef-building coral. Mar. Biotechnol..

[45] Bessette P.H., Daugherty P.S. (2004). Flow cytometric screening of cDNA expression libraries for fluorescent proteins. Biotechnol. Prog..

[46] Labas Y.A., Gurskaya N.G., Yanushevich Y.G., Fradkov A.F., Lukyanov K.A., Lukyanov S.A., Matz M.V. (2002). Diversity and evolution of the green fluorescent protein family. Proc. Natl. Acad. Sci. USA.

[47] Fradkov A.F., Chen Y., Ding L., Barsova E.V., Matz M.V., Lukyanov S.A. (2000). Novel fluorescent protein from *Discosoma* coral and its mutants possesses a unique far-red fluorescence. FEBS Lett..

[48] Wiedenmann J., Schenk A., Röcker C., Girod A., Spindler K.-D., Nienhaus G.U. (2002). A far-red fluorescent protein with fast maturation and reduced oligomerization tendency from *Entacmaea quadricolor* (Anthozoa, Actinaria). Proc. Natl. Acad. Sci. USA.

[49] Gurskaya N.G., Fradkov A.F., Terskikh A., Matz M.V., Labas Y.A., Martynov V.I., Yanushevich Y.G., Lukyanov K.A., Lukyanov S.A. (2001). GFP-like chromoproteins as a source of far-red fluorescent proteins. FEBS Lett..

[50] Masuda H., Takenaka Y., Yamaguchi A., Nishikawa S., Mizuno H. (2006). A novel yellowish-green fluorescent protein from the marine copepod, Chiridius poppei, and its use as a reporter protein in HeLa cells. Gene.

[51] Shagin D.A., Barsova E.V., Yanushevich Y.G., Fradkov A.F., Lukyanov K.A., Labas Y.A., Semenova T.N., Ugalde J.A., Meyers A., Nunez J.M. (2004). GFP-like proteins as ubiquitous metazoan superfamily: Evolution of functional features and structural complexity. Mol. Biol. Evol..

[52] Notung. http://www.cs.cmu.edu/~durand/Notung/.

[53] Chen K., Durand D., Farach-Colton M. (2000). NOTUNG: A program for dating gene duplications and optimizing gene family trees. J. Comput. Biol..

[54] Skinner M.E., Uzilov A.V., Stein L.D., Mungall C.J., Holmes I.H. (2009). JBrowse: A next-generation genome browser. Genome Res..

[55] Jones P., Binns D., Chang H.-Y., Fraser M., Li W., McAnulla C., McWilliam H., Maslen J., Mitchell A., Nuka G. (2014). InterProScan 5: Genome-scale protein function classification. Bioinformatics.

[56] RefSeq: NCBI Reference Sequence Database. https://www.ncbi.nlm.nih.gov/refseq/.

[57] Ovcharenko I., Loots G., Hardison R., Miller W., Stubbs L. (2004). zPicture: Dynamic alignment and visualization tool for analyzing conservation profiles. Genome Res..

[58] zPicture: Dynamic Blastz Alignment Visualization. https://zpicture.dcode.org/.

[59] Olde K., Jarvis I., Uličný D., Pearce M.A., Trabucho-Alexandre J., Čech S., Gröcke D.R., Laurin J., Švábenická L., Tocher B.A. (2015). Geochemical and palynological sea-level proxies in hemipelagic sediments: A critical assessment from the Upper Cretaceous of the Czech Republic. Palaeogeogr. Palaeoclimatol. Palaeoecol..

[60] Cramwinckel M.J., Huber M., Kocken I.J., Agnini C., Bijl P.K., Bohaty S.M., Frieling J., Goldner A., Hilgen F.J., Kip E.L. (2018). Synchronous tropical and polar temperature evolution in the Eocene. Nature.

[61] Prag S., Adams J.C. (2003). Molecular phylogeny of the kelch-repeat superfamily reveals an expansion of BTB/kelch proteins in animals. BMC Bioinform..

[62] Tautz D., Domazet-Lošo T. (2011). The evolutionary origin of orphan genes. Nat. Rev. Genet..

[63] Gittins J.R., D’Angelo C., Oswald F., Edwards R.J., Wiedenmann J. (2015). Fluorescent protein-mediated colour polymorphism in reef corals: Multicopy genes extend the adaptation/acclimatization potential to variable light environments. Mol. Ecol..

