# Supplementary material for: Expansion and Diversification of Fluorescent Protein Genes in Fifteen Acropora Species during the Evolution of Acroporid Corals
Source: Genes (Basel). 2021 Mar 11;12(3):397. doi: 10.3390/genes12030397 (PMC8001845; doi:10.3390/genes12030397)
Supplement: Supplementary file 1 [file genes-12-00397-s001.pdf]

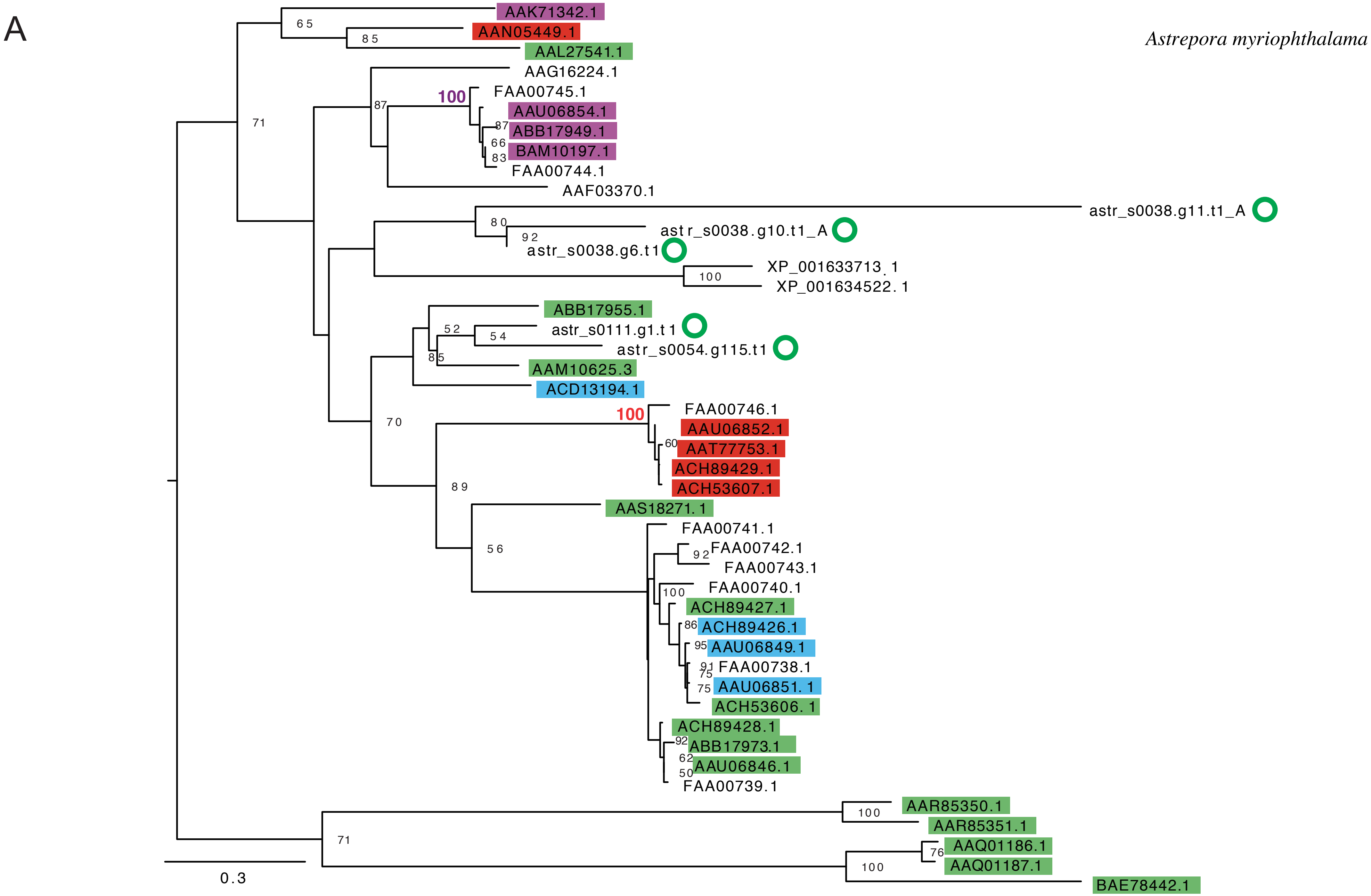

Figure S1. FP molecular phylogeny of 15 species of *Acropora*, two *Montipora*, and one *Astreopora* in the family Acroporidae.
FP molecular phylogenic trees in *Astreopora* (A), two *Montipora* (B-C), and 15 species of *Acropora* (D-R) were generated separately for each taxon. Confirmed fluorescent protein colors are indicated by highlighting (See also Table 1). Predicted GFP/CFP, RFP, and Chrp proteins are marked with green, red and purple circles, respectively. Bootstrap values ≥50% are indicated at each node. RFPs and Chrps clades with high bootstrap values (≥90%) are indicated by red or purple numbers, respectively. Protein IDs are labeled with the first letter of the generic name and a three-letter abbreviation of specific epithet (e.g., aten for *Acropora tenuis*), except for astr for *Astreopora myriophthalam*.

B

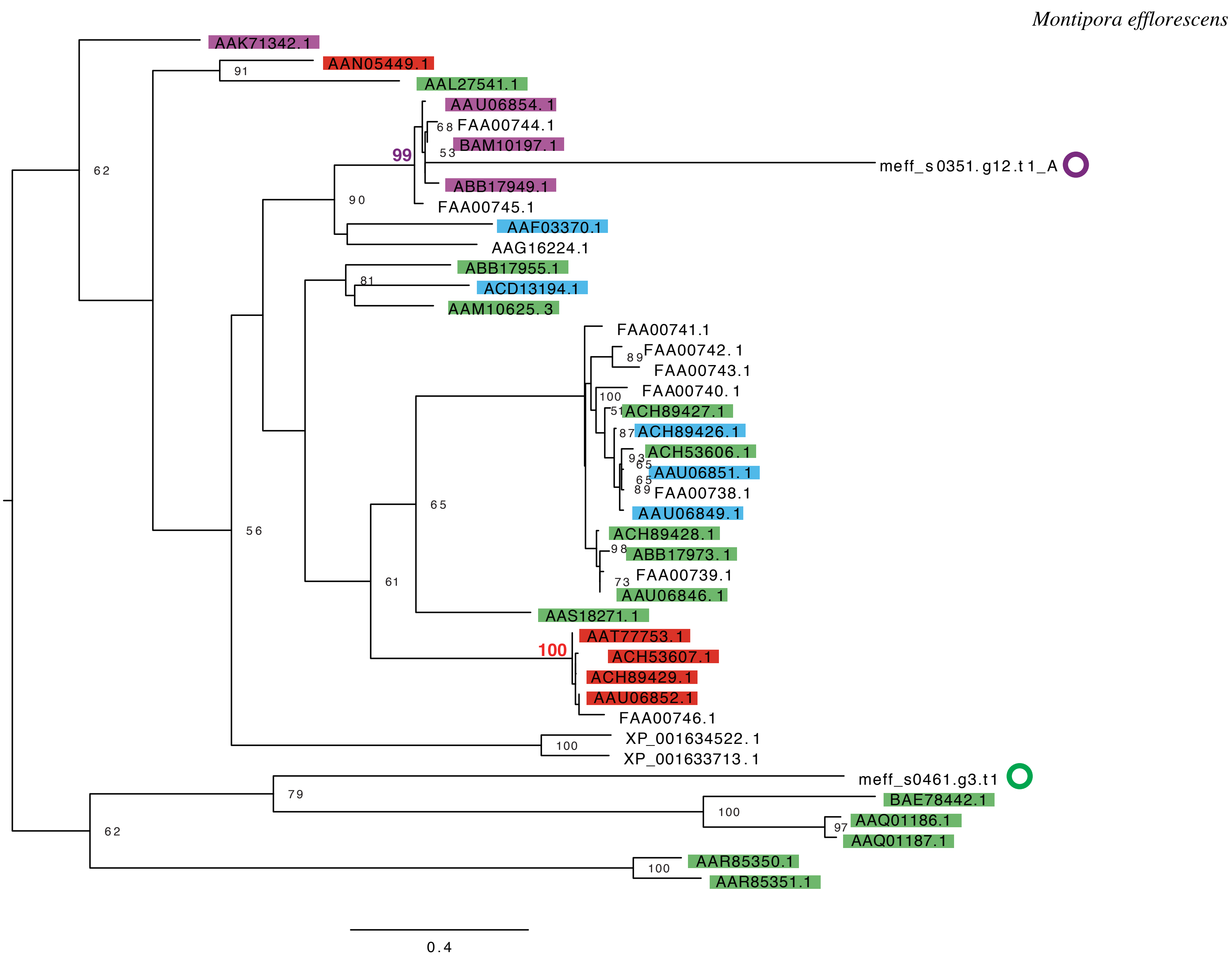
Montipora efflorescens
AAK71342.1
AAN05449.1
91
AAL27541.1
AAU06854.1
68 FAA00744.1
BAM10197.1
53
99
meff_s0351.g12.t1_A
ABB17949.1
90
FAA00745.1
AAF03370.1
AAG16224.1
62
ABB17955.1
81
ACD13194.1
AAM10625.3
FAA00741.1
89 FAA00742.1
FAA00743.1
FAA00740.1
100
51 ACH89427.1
87 ACH89426.1
93 ACH53606.1
65
AAU06851.1
65
89 FAA00738.1
AAU06849.1
65
ACH89428.1
98 ABB17973.1
73 FAA00739.1
AAU06846.1
56
61
AAS18271.1
AAT77753.1
100
ACH53607.1
ACH89429.1
AAU06852.1
FAA00746.1
100 XP_001634522.1
XP_001633713.1
meff_s0461.g3.t1
79
BAE78442.1
100
97 AAQ01186.1
AAQ01187.1
62
100 AAR85350.1
AAR85351.1
0.4

C

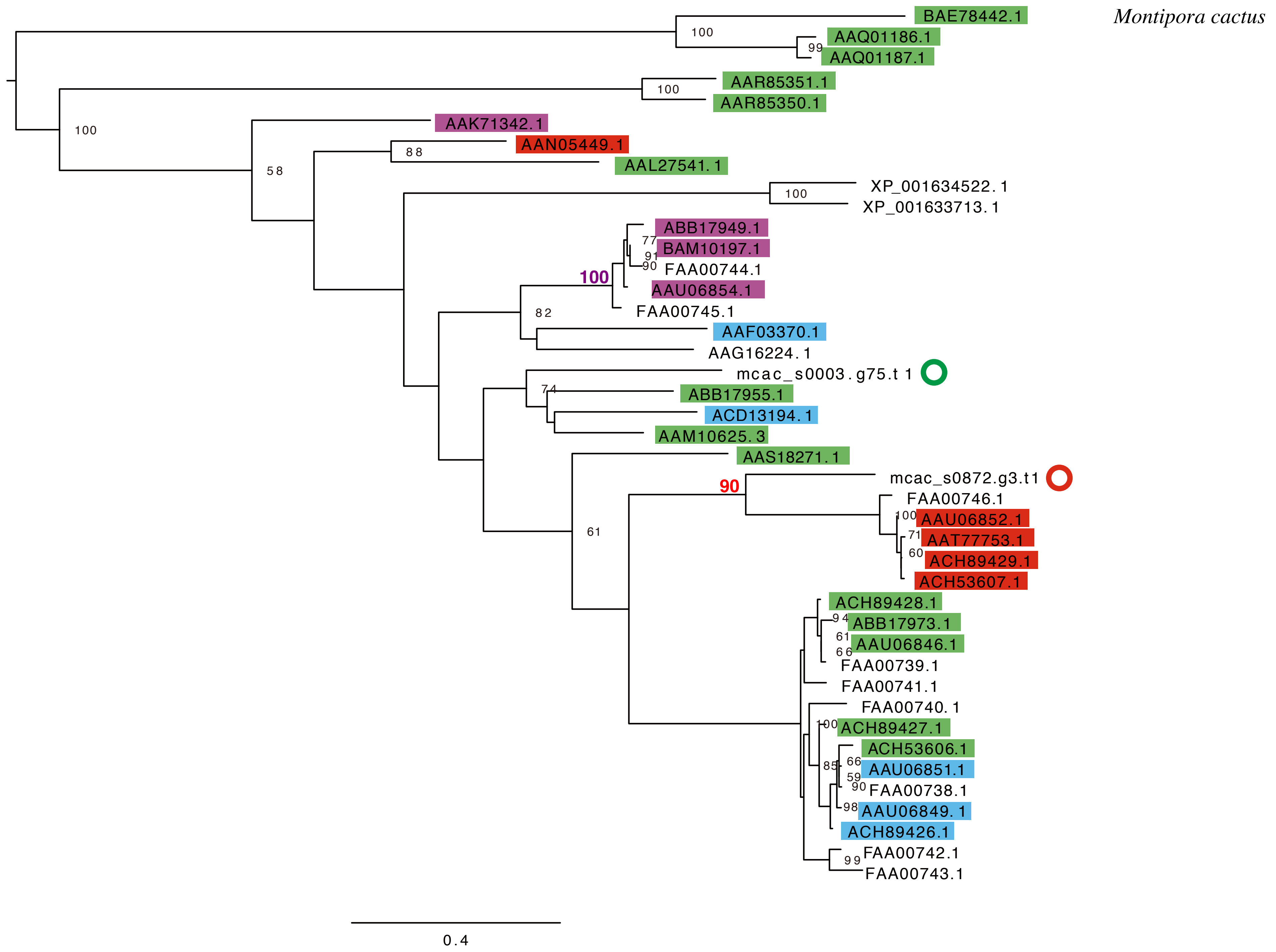

Montipora cactus
BAE78442.1
AAQ01186.1
AAQ01187.1
AAR85351.1
AAR85350.1
AAK71342.1
AAN05449.1
AAL27541.1
XP_001634522.1
XP_001633713.1
ABB17949.1
BAM10197.1
FAA00744.1
AAU06854.1
FAA00745.1
AAF03370.1
AAG16224.1
mcac_s0003.g75.t1
ABB17955.1
ACD13194.1
AAM10625.3
AAS18271.1
mcac_s0872.g3.t1
FAA00746.1
AAU06852.1
AAT77753.1
ACH89429.1
ACH53607.1
ACH89428.1
ABB17973.1
AAU06846.1
FAA00739.1
FAA00741.1
FAA00740.1
ACH89427.1
ACH53606.1
AAU06851.1
FAA00738.1
AAU06849.1
ACH89426.1
FAA00742.1
FAA00743.1
0.4

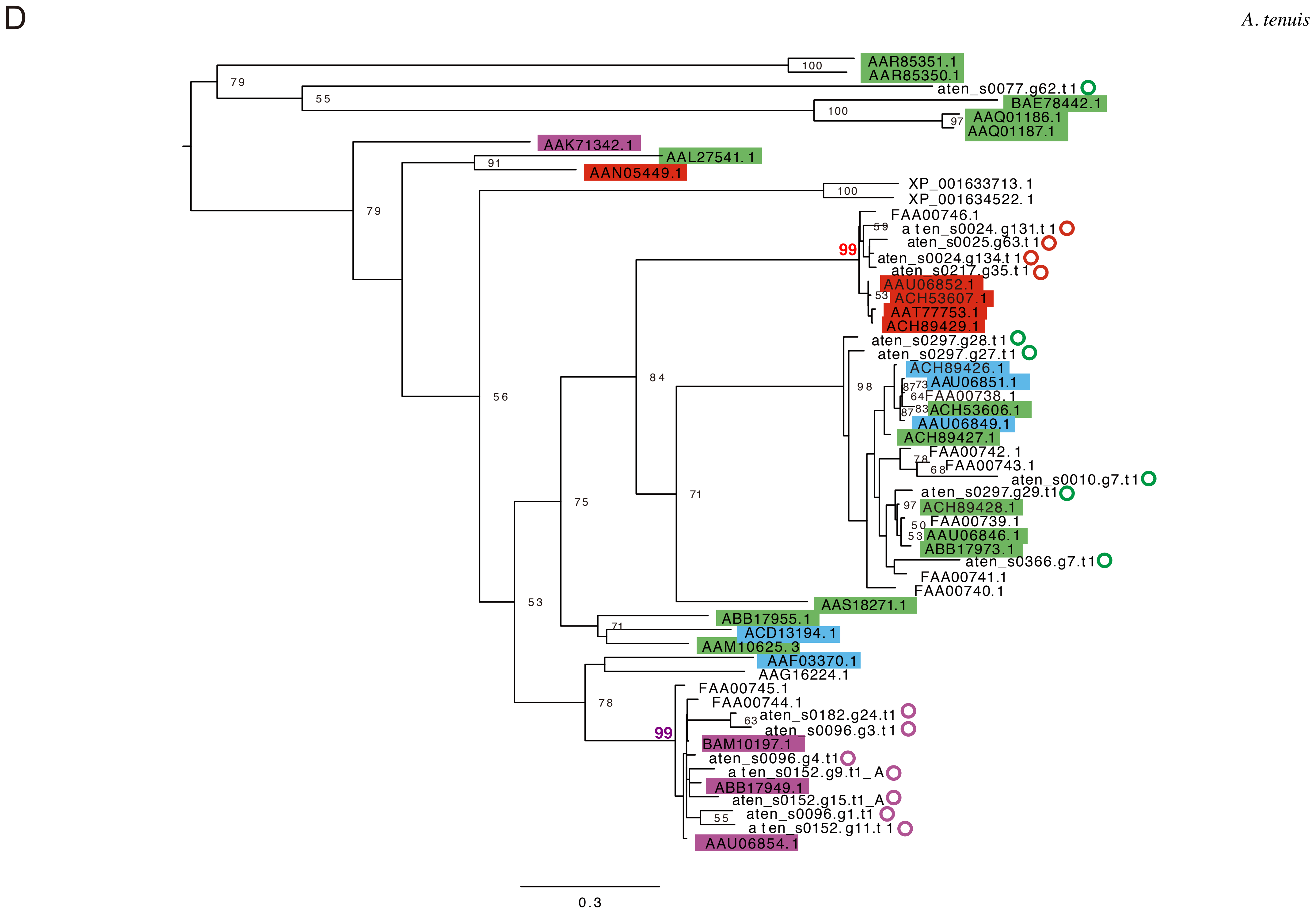
D
A. tenuis
AAR85351.1
AAR85350.1
aten_s0077.g62.t1
BAE78442.1
AAQ01186.1
AAQ01187.1
AAK71342.1
AAL27541.1
AAN05449.1
XP_001633713.1
XP_001634522.1
FAA00746.1
aten_s0024.g131.t1
aten_s0025.g63.t1
aten_s0024.g134.t1
aten_s0217.g35.t1
AAU06852.1
ACH53607.1
AAT77753.1
ACH89429.1
aten_s0297.g28.t1
aten_s0297.g27.t1
ACH89426.1
AAU06851.1
FAA00738.1
ACH53606.1
AAU06849.1
ACH89427.1
FAA00742.1
FAA00743.1
aten_s0010.g7.t1
aten_s0297.g29.t1
ACH89428.1
FAA00739.1
AAU06846.1
ABB17973.1
aten_s0366.g7.t1
FAA00741.1
FAA00740.1
AAS18271.1
ABB17955.1
ACD13194.1
AAM10625.3
AAF03370.1
AAG16224.1
FAA00745.1
FAA00744.1
aten_s0182.g24.t1
aten_s0096.g3.t1
BAM10197.1
aten_s0096.g4.t1
aten_s0152.g9.t1_A
ABB17949.1
aten_s0152.g15.t1_A
aten_s0096.g1.t1
aten_s0152.g11.t1
AAU06854.1
0.3

E

A. yongei

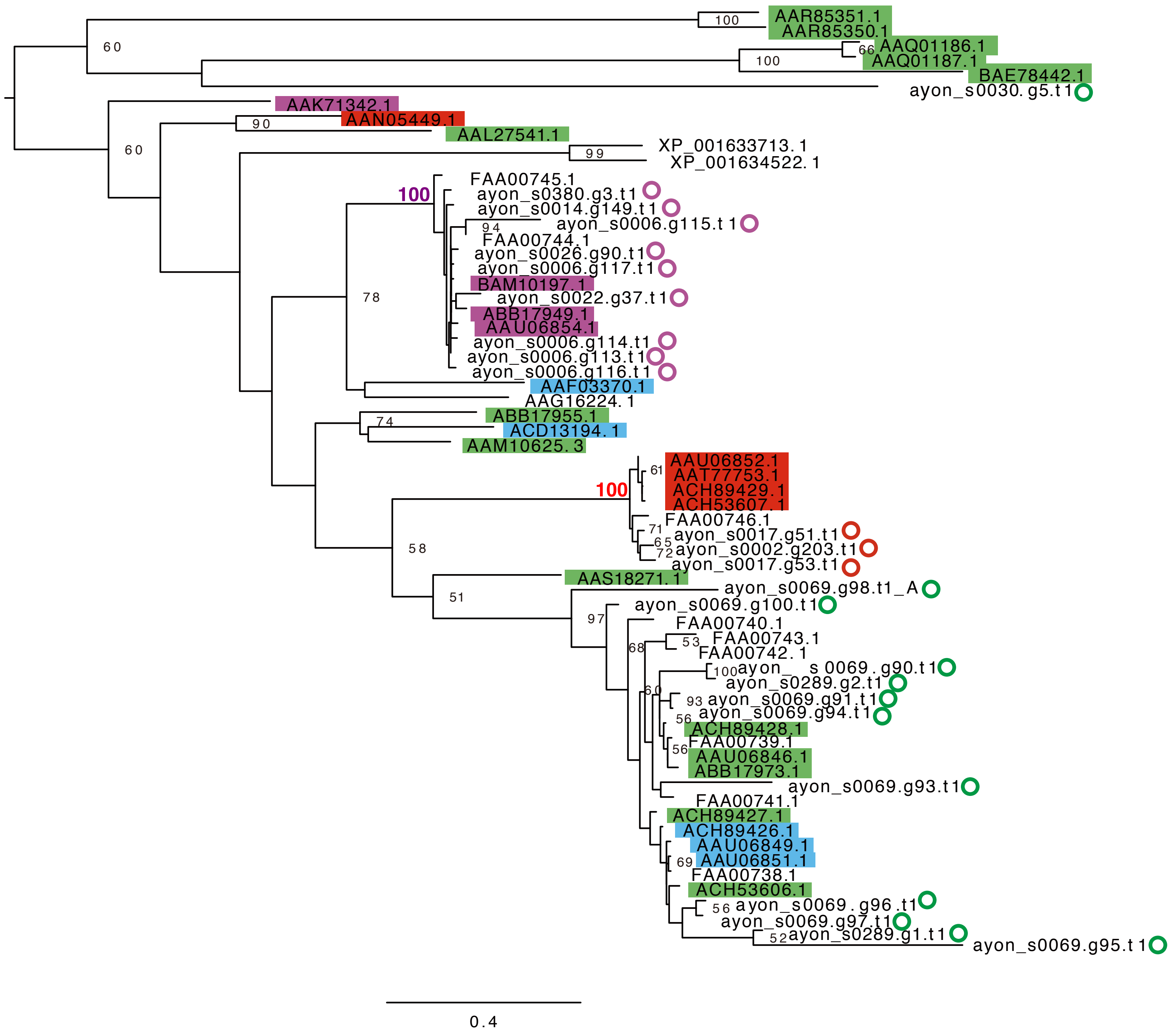

AAR85351.1
AAR85350.1
AAQ01186.1
AAQ01187.1
BAE78442.1
ayon_s0030.g5.t1
AAK71342.1
AAN05449.1
AAL27541.1
XP_001633713.1
XP_001634522.1
FAA00745.1
ayon_s0380.g3.t1
ayon_s0014.g149.t1
ayon_s0006.g115.t1
FAA00744.1
ayon_s0026.g90.t1
ayon_s0006.g117.t1
BAM10197.1
ayon_s0022.g37.t1
ABB17949.1
AAU06854.1
ayon_s0006.g114.t1
ayon_s0006.g113.t1
ayon_s0006.g116.t1
AAF03370.1
AAG16224.1
ABB17955.1
ACD13194.1
AAM10625.3
AAU06852.1
AAT77753.1
ACH89429.1
ACH53607.1
FAA00746.1
ayon_s0017.g51.t1
ayon_s0002.g203.t1
ayon_s0017.g53.t1
AAS18271.1
ayon_s0069.g98.t1_A
ayon_s0069.g100.t1
FAA00740.1
FAA00743.1
FAA00742.1
ayon_s0069.g90.t1
ayon_s0289.g2.t1
ayon_s0069.g91.t1
ayon_s0069.g94.t1
ACH89428.1
FAA00739.1
AAU06846.1
ABB17973.1
ayon_s0069.g93.t1
FAA00741.1
ACH89427.1
ACH89426.1
AAU06849.1
AAU06851.1
FAA00738.1
ACH53606.1
ayon_s0069.g96.t1
ayon_s0069.g97.t1
ayon_s0289.g1.t1
ayon_s0069.g95.t1
0.4

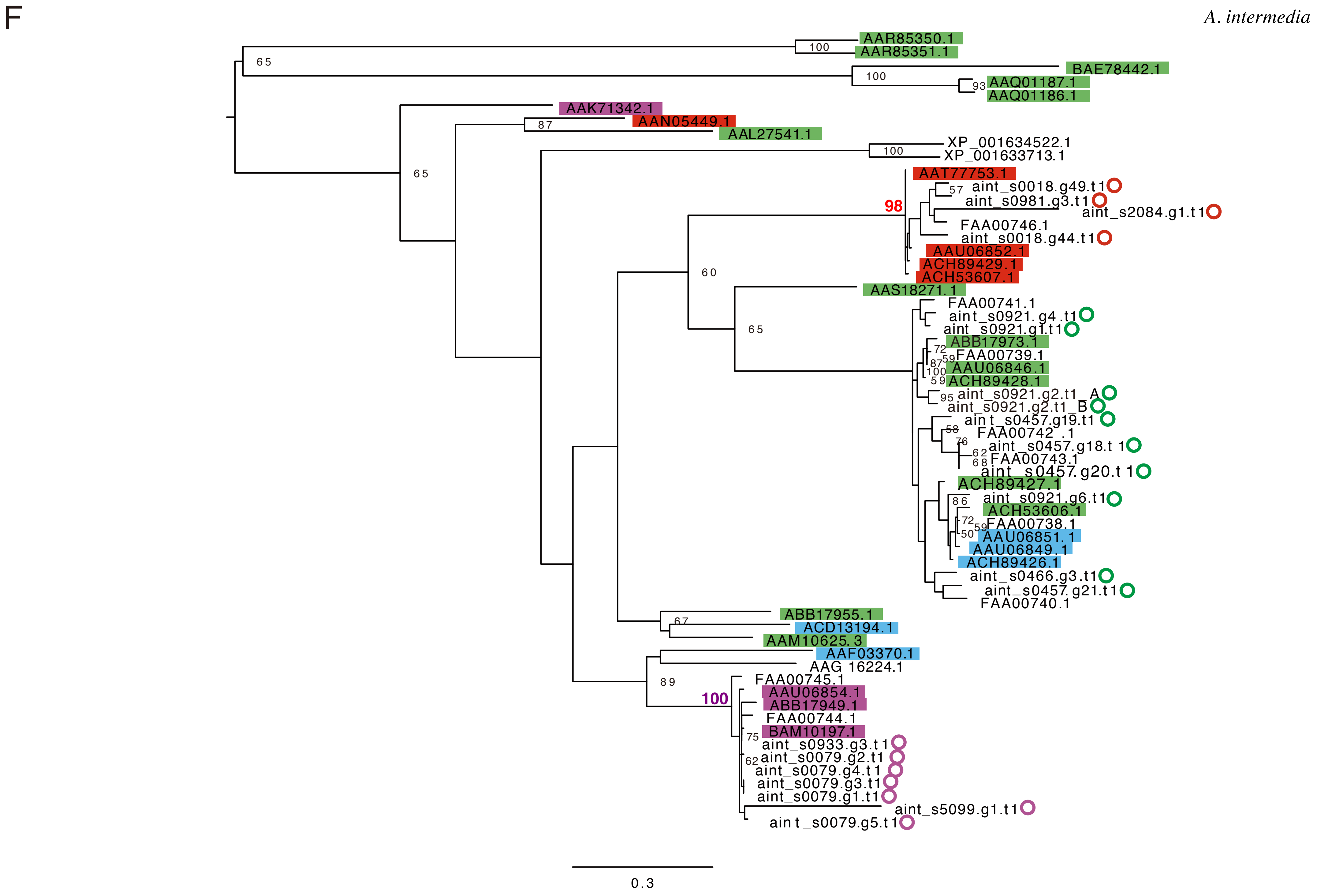
F
A. intermedia
AAR85350.1
AAR85351.1
BAE78442.1
AAQ01187.1
AAQ01186.1
AAK71342.1
AAN05449.1
AAL27541.1
XP_001634522.1
XP_001633713.1
AAT77753.1
aint_s0018.g49.t1
aint_s0981.g3.t1
aint_s2084.g1.t1
FAA00746.1
aint_s0018.g44.t1
AAU06852.1
ACH89429.1
ACH53607.1
AAS18271.1
FAA00741.1
aint_s0921.g4.t1
aint_s0921.g1.t1
ABB17973.1
FAA00739.1
AAU06846.1
ACH89428.1
aint_s0921.g2.t1_A
aint_s0921.g2.t1_B
aint_s0457.g19.t1
FAA00742.1
aint_s0457.g18.t1
FAA00743.1
aint_s0457.g20.t1
ACH89427.1
aint_s0921.g6.t1
ACH53606.1
FAA00738.1
AAU06851.1
AAU06849.1
ACH89426.1
aint_s0466.g3.t1
aint_s0457.g21.t1
FAA00740.1
ABB17955.1
ACD13194.1
AAM10625.3
AAF03370.1
AAG16224.1
FAA00745.1
AAU06854.1
ABB17949.1
FAA00744.1
BAM10197.1
aint_s0933.g3.t1
aint_s0079.g2.t1
aint_s0079.g4.t1
aint_s0079.g3.t1
aint_s0079.g1.t1
aint_s5099.g1.t1
aint_s0079.g5.t1
65
100
100
93
65
87
100
98
57
60
65
72
59
87
100
59
95
58
76
62
68
86
72
59
50
67
89
100
75
62
0.3

G

A. gemmifera

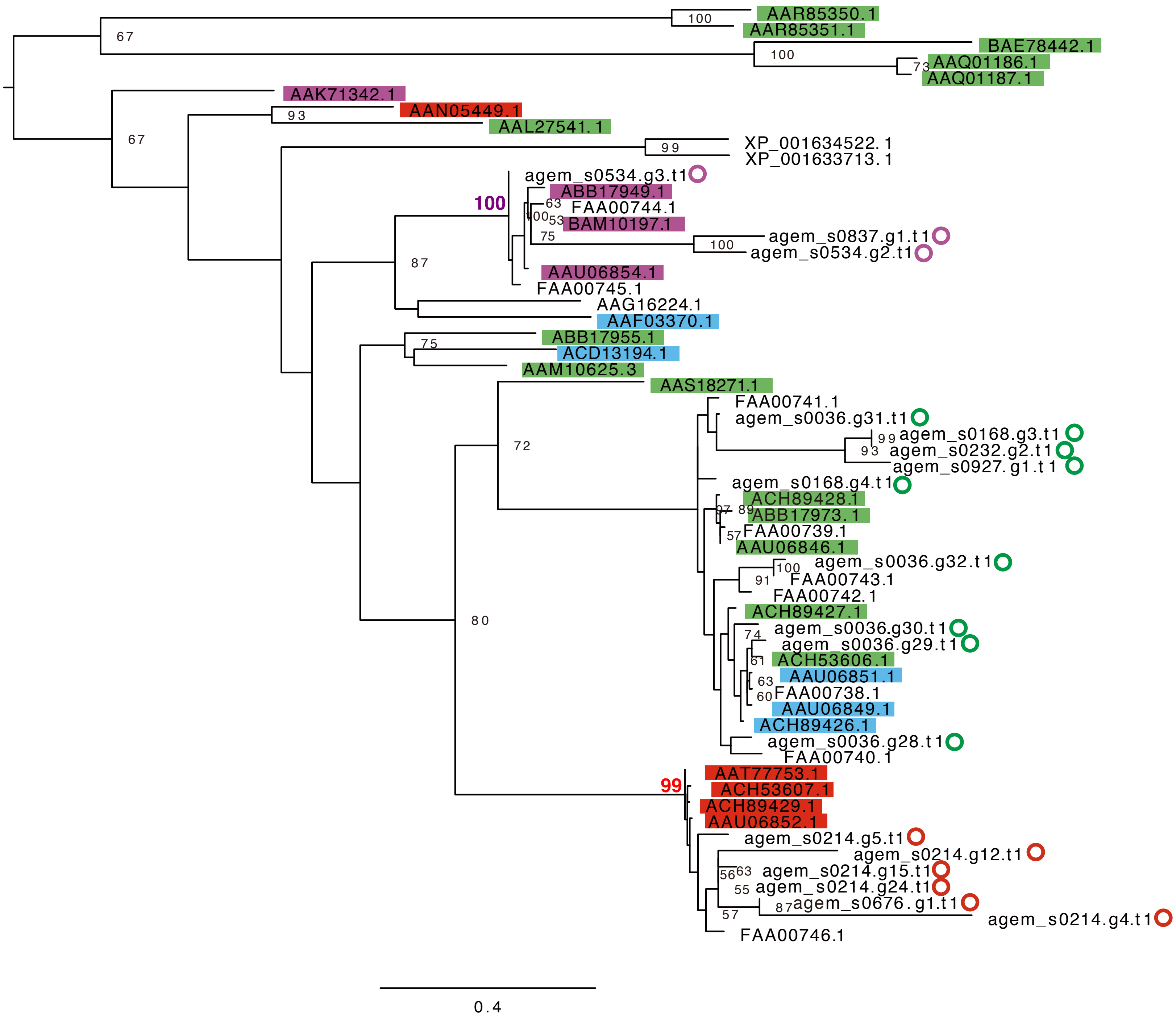

H

A. awi

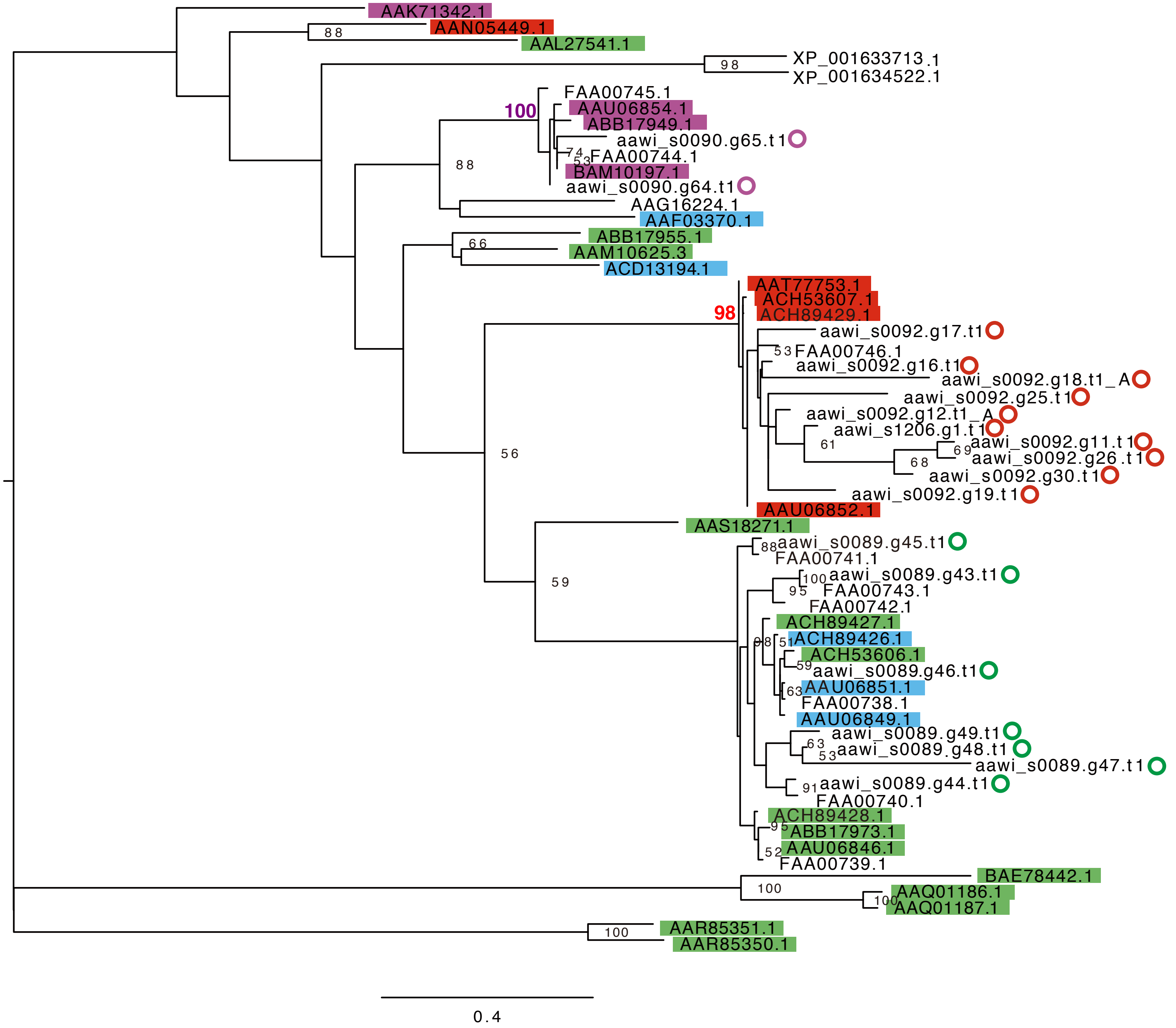
AAK71342.1
88
AAN05449.1
AAL27541.1
98
XP_001633713.1
XP_001634522.1
FAA00745.1
100
AAU06854.1
ABB17949.1
aawi_s0090.g65.t1
74
53
FAA00744.1
88
BAM10197.1
aawi_s0090.g64.t1
AAG16224.1
AAF03370.1
ABB17955.1
66
AAM10625.3
ACD13194.1
AAT77753.1
ACH53607.1
98
ACH89429.1
aawi_s0092.g17.t1
53
FAA00746.1
aawi_s0092.g16.t1
aawi_s0092.g18.t1_A
aawi_s0092.g25.t1
aawi_s0092.g12.t1_A
aawi_s1206.g1.t1
61
69
aawi_s0092.g11.t1
68
aawi_s0092.g26.t1
56
aawi_s0092.g30.t1
aawi_s0092.g19.t1
AAU06852.1
AAS18271.1
88
aawi_s0089.g45.t1
FAA00741.1
100
aawi_s0089.g43.t1
95
FAA00743.1
FAA00742.1
59
ACH89427.1
98
51
ACH89426.1
ACH53606.1
59
aawi_s0089.g46.t1
63
AAU06851.1
FAA00738.1
AAU06849.1
aawi_s0089.g49.t1
63
53
aawi_s0089.g48.t1
aawi_s0089.g47.t1
91
aawi_s0089.g44.t1
FAA00740.1
ACH89428.1
95
ABB17973.1
52
AAU06846.1
FAA00739.1
BAE78442.1
100
100
AAQ01186.1
AAQ01187.1
100
AAR85351.1
AAR85350.1
0.4

I

A. florida

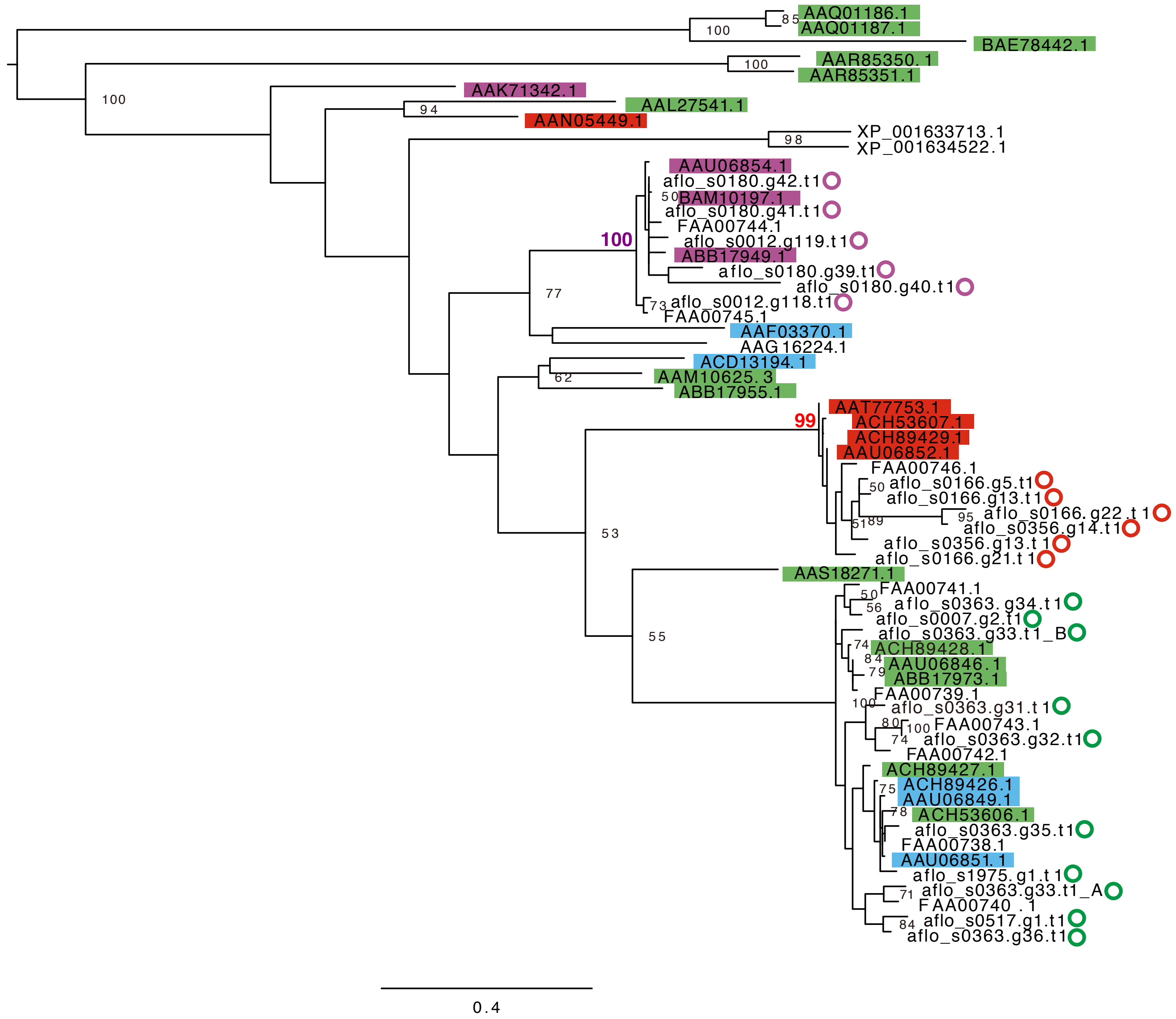

AAQ01186.1
AAQ01187.1
BAE78442.1
AAR85350.1
AAR85351.1
AAK71342.1
AAL27541.1
AAN05449.1
XP_001633713.1
XP_001634522.1
AAU06854.1
aflo_s0180.g42.t1
BAM10197.1
aflo_s0180.g41.t1
FAA00744.1
aflo_s0012.g119.t1
ABB17949.1
aflo_s0180.g39.t1
aflo_s0180.g40.t1
aflo_s0012.g118.t1
FAA00745.1
AAF03370.1
AAG16224.1
ACD13194.1
AAM10625.3
ABB17955.1
AAT77753.1
ACH53607.1
ACH89429.1
AAU06852.1
FAA00746.1
aflo_s0166.g5.t1
aflo_s0166.g13.t1
aflo_s0166.g22.t1
aflo_s0356.g14.t1
aflo_s0356.g13.t1
aflo_s0166.g21.t1
AAS18271.1
FAA00741.1
aflo_s0363.g34.t1
aflo_s0007.g2.t1
aflo_s0363.g33.t1_B
ACH89428.1
AAU06846.1
ABB17973.1
FAA00739.1
aflo_s0363.g31.t1
FAA00743.1
aflo_s0363.g32.t1
FAA00742.1
ACH89427.1
ACH89426.1
AAU06849.1
ACH53606.1
aflo_s0363.g35.t1
FAA00738.1
AAU06851.1
aflo_s1975.g1.t1
aflo_s0363.g33.t1_A
FAA00740.1
aflo_s0517.g1.t1
aflo_s0363.g36.t1
0.4

J

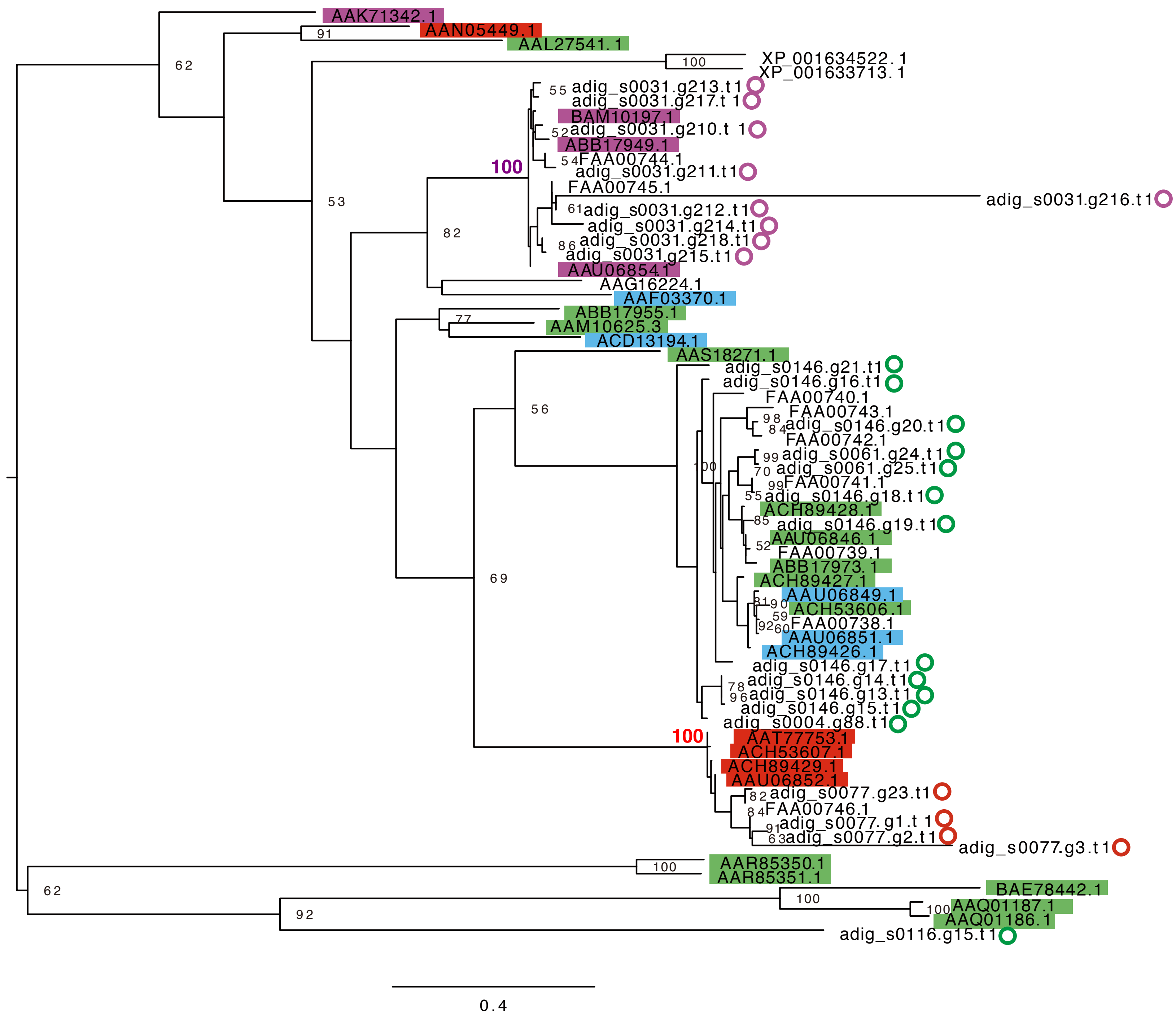
A. digitifera
AAK71342.1
AAN05449.1
AAL27541.1
XP_001634522.1
XP_001633713.1
adig_s0031.g213.t1
adig_s0031.g217.t1
BAM10197.1
adig_s0031.g210.t1
ABB17949.1
FAA00744.1
adig_s0031.g211.t1
FAA00745.1
adig_s0031.g216.t1
adig_s0031.g212.t1
adig_s0031.g214.t1
adig_s0031.g218.t1
adig_s0031.g215.t1
AAU06854.1
AAG16224.1
AAF03370.1
ABB17955.1
AAM10625.3
ACD13194.1
AAS18271.1
adig_s0146.g21.t1
adig_s0146.g16.t1
FAA00740.1
FAA00743.1
adig_s0146.g20.t1
FAA00742.1
adig_s0061.g24.t1
adig_s0061.g25.t1
FAA00741.1
adig_s0146.g18.t1
ACH89428.1
adig_s0146.g19.t1
AAU06846.1
FAA00739.1
ABB17973.1
ACH89427.1
AAU06849.1
ACH53606.1
FAA00738.1
AAU06851.1
ACH89426.1
adig_s0146.g17.t1
adig_s0146.g14.t1
adig_s0146.g13.t1
adig_s0146.g15.t1
adig_s0004.g88.t1
AAT77753.1
ACH53607.1
ACH89429.1
AAU06852.1
adig_s0077.g23.t1
FAA00746.1
adig_s0077.g1.t1
adig_s0077.g2.t1
adig_s0077.g3.t1
AAR85350.1
AAR85351.1
BAE78442.1
AAQ01187.1
AAQ01186.1
adig_s0116.g15.t1
0.4

K

A. nasuta

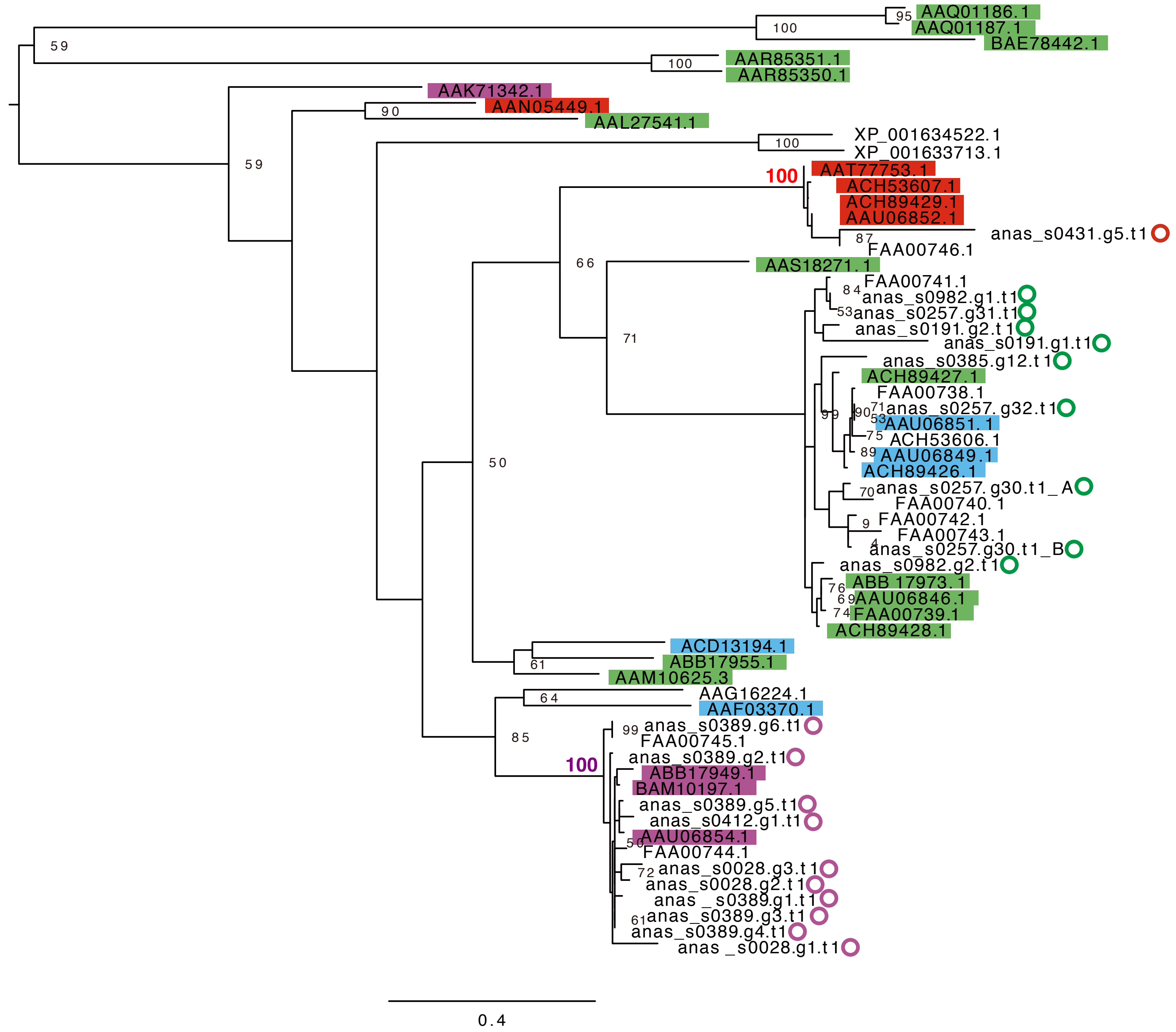

AAQ01186.1
AAQ01187.1
BAE78442.1
AAR85351.1
AAR85350.1
AAK71342.1
AAN05449.1
AAL27541.1
XP_001634522.1
XP_001633713.1
AAT77753.1
ACH53607.1
ACH89429.1
AAU06852.1
anas_s0431.g5.t1
FAA00746.1
AAS18271.1
FAA00741.1
anas_s0982.g1.t1
anas_s0257.g31.t1
anas_s0191.g2.t1
anas_s0191.g1.t1
anas_s0385.g12.t1
ACH89427.1
FAA00738.1
anas_s0257.g32.t1
AAU06851.1
ACH53606.1
AAU06849.1
ACH89426.1
anas_s0257.g30.t1_A
FAA00740.1
FAA00742.1
FAA00743.1
anas_s0257.g30.t1_B
anas_s0982.g2.t1
ABB17973.1
AAU06846.1
FAA00739.1
ACH89428.1
ACD13194.1
ABB17955.1
AAM10625.3
AAG16224.1
AAF03370.1
anas_s0389.g6.t1
FAA00745.1
anas_s0389.g2.t1
ABB17949.1
BAM10197.1
anas_s0389.g5.t1
anas_s0412.g1.t1
AAU06854.1
FAA00744.1
anas_s0028.g3.t1
anas_s0028.g2.t1
anas_s0389.g1.t1
anas_s0389.g3.t1
anas_s0389.g4.t1
anas_s0028.g1.t1
95
100
59
100
90
59
100
100
87
66
71
84
53
99
90
71
53
75
89
70
9
4
76
69
74
50
61
64
85
100
99
50
72
61
0.4

L

A. microphthalma

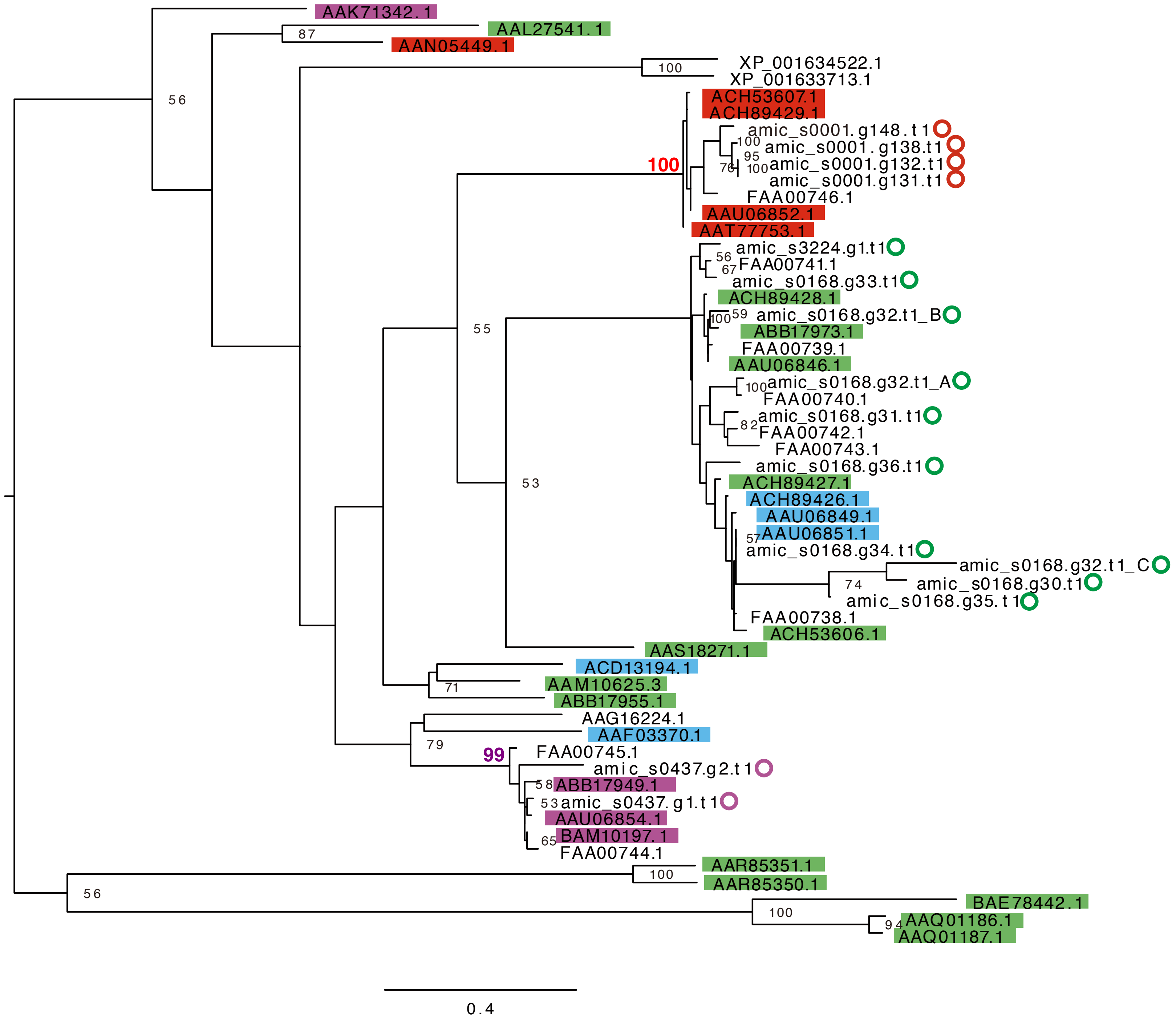

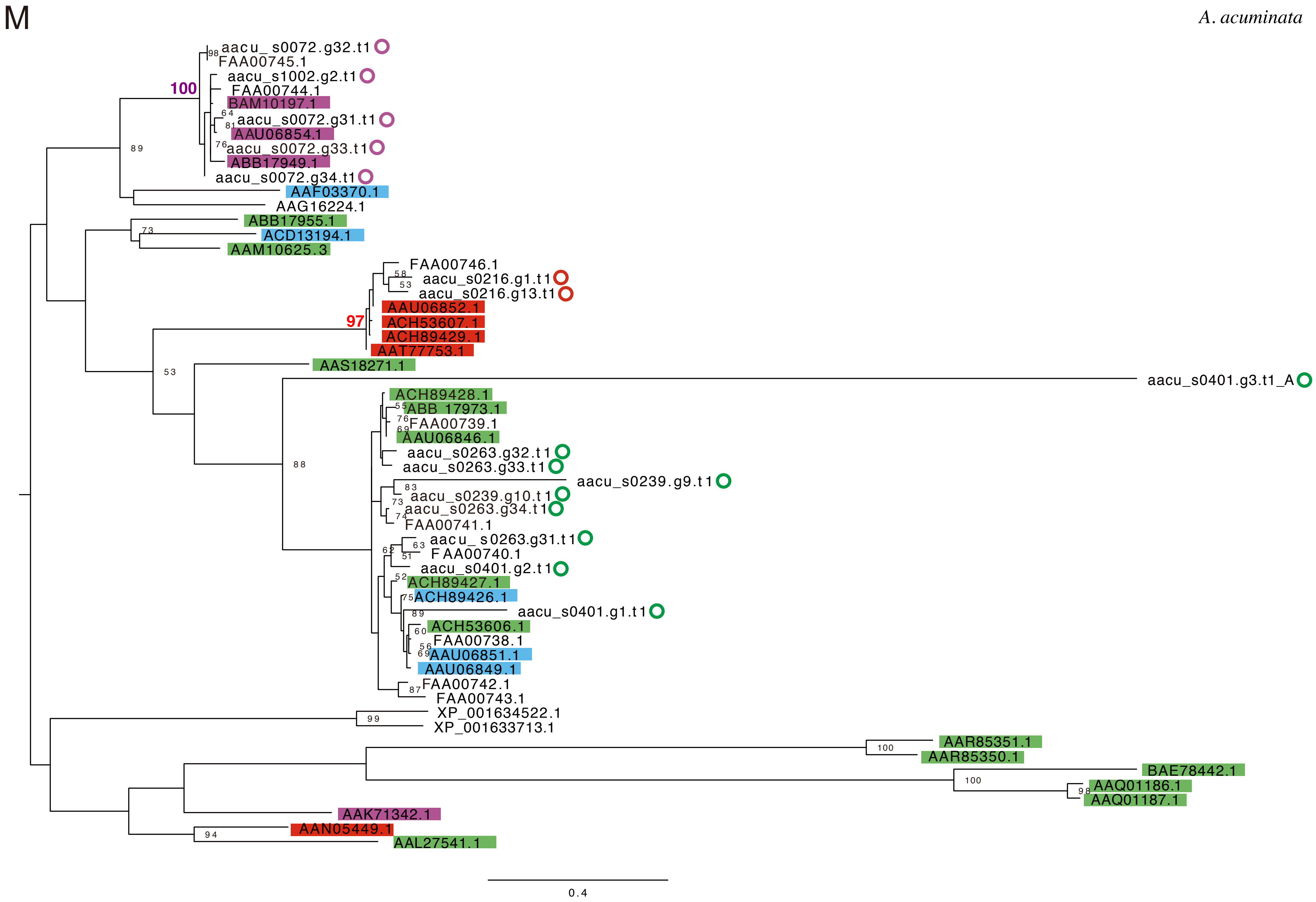
M
A. acuminata
aacu_s0072.g32.t1
FAA00745.1
aacu_s1002.g2.t1
FAA00744.1
BAM10197.1
aacu_s0072.g31.t1
AAU06854.1
aacu_s0072.g33.t1
ABB17949.1
aacu_s0072.g34.t1
AAF03370.1
AAG16224.1
ABB17955.1
ACD13194.1
AAM10625.3
FAA00746.1
aacu_s0216.g1.t1
aacu_s0216.g13.t1
AAU06852.1
ACH53607.1
ACH89429.1
AAT77753.1
AAS18271.1
aacu_s0401.g3.t1_A
ACH89428.1
ABB 17973.1
FAA00739.1
AAU06846.1
aacu_s0263.g32.t1
aacu_s0263.g33.t1
aacu_s0239.g9.t1
aacu_s0239.g10.t1
aacu_s0263.g34.t1
FAA00741.1
aacu_s0263.g31.t1
FAA00740.1
aacu_s0401.g2.t1
ACH89427.1
ACH89426.1
aacu_s0401.g1.t1
ACH53606.1
FAA00738.1
AAU06851.1
AAU06849.1
FAA00742.1
FAA00743.1
XP_001634522.1
XP_001633713.1
AAR85351.1
AAR85350.1
BAE78442.1
AAQ01186.1
AAQ01187.1
AAK71342.1
AAN05449.1
AAL27541.1
0.4

N

A. echinata

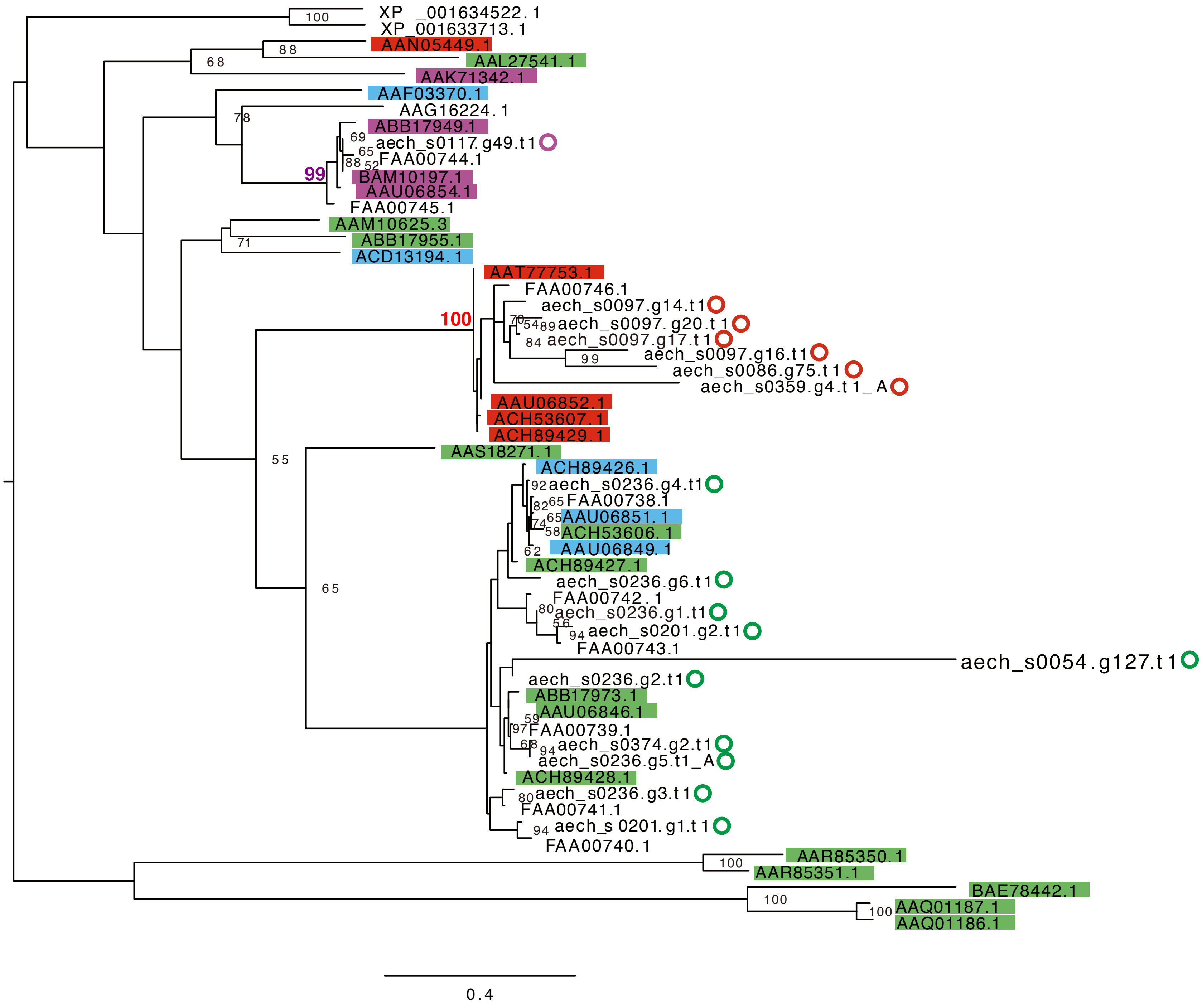
XP _001634522. 1
XP_001633713. 1
AAN05449.1
AAL27541. 1
AAK71342. 1
AAF03370.1
AAG16224. 1
ABB17949.1
aech_s0117.g49.t1
FAA00744.1
BAM10197.1
AAU06854.1
FAA00745.1
AAM10625.3
ABB17955.1
ACD13194. 1
AAT77753.1
FAA00746.1
aech_s0097.g14.t1
aech_s0097. g20.t 1
aech_s0097.g17.t 1
aech_s0097.g16.t1
aech_s0086.g75.t 1
aech_s0359.g4.t 1_ A
AAU06852.1
ACH53607.1
ACH89429.1
AAS18271.1
ACH89426.1
aech_s0236.g4.t1
FAA00738.1
AAU06851. 1
ACH53606. 1
AAU06849. 1
ACH89427.1
aech_s0236.g6.t1
FAA00742. 1
aech_s0236.g1.t1
aech_s0201.g2.t1
FAA00743.1
aech_s0054.g127.t1
aech_s0236.g2.t1
ABB17973.1
AAU06846.1
FAA00739.1
aech_s0374.g2.t1
aech_s0236.g5.t1_A
ACH89428.1
aech_s0236.g3.t1
FAA00741.1
aech_s 0201. g1.t 1
FAA00740. 1
AAR85350. 1
AAR85351.1
BAE78442.1
AAQ01187.1
AAQ01186.1
0.4

O

A. muricata

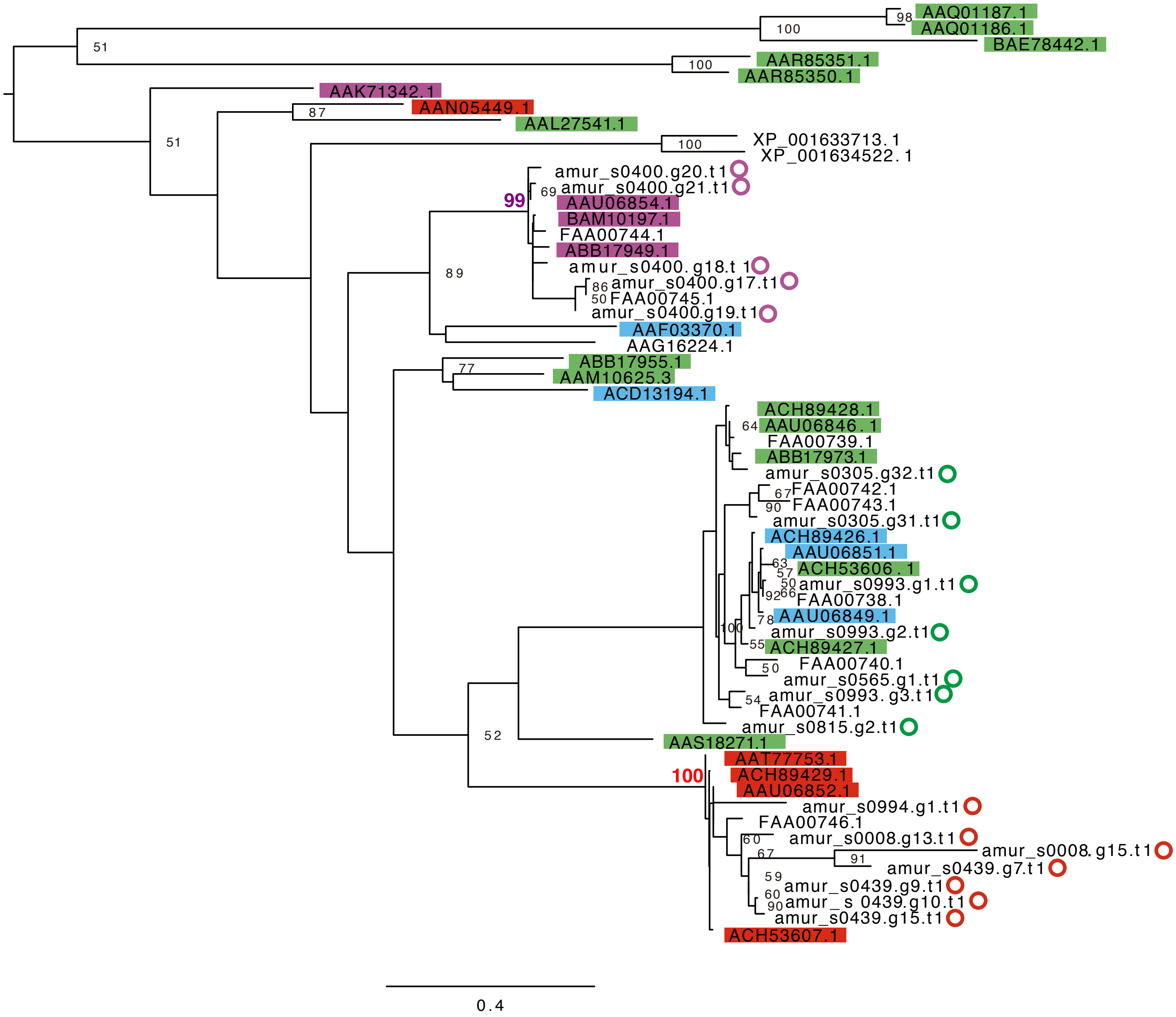

AAQ01187.1
AAQ01186.1
BAE78442.1
AAR85351.1
AAR85350.1
AAK71342.1
AAN05449.1
AAL27541.1
XP_001633713.1
XP_001634522.1
amur_s0400.g20.t1
amur_s0400.g21.t1
AAU06854.1
BAM10197.1
FAA00744.1
ABB17949.1
amur_s0400.g18.t1
amur_s0400.g17.t1
FAA00745.1
amur_s0400.g19.t1
AAF03370.1
AAG16224.1
ABB17955.1
AAM10625.3
ACD13194.1
ACH89428.1
AAU06846.1
FAA00739.1
ABB17973.1
amur_s0305.g32.t1
FAA00742.1
FAA00743.1
amur_s0305.g31.t1
ACH89426.1
AAU06851.1
ACH53606.1
amur_s0993.g1.t1
FAA00738.1
AAU06849.1
amur_s0993.g2.t1
ACH89427.1
FAA00740.1
amur_s0565.g1.t1
amur_s0993.g3.t1
FAA00741.1
amur_s0815.g2.t1
AAS18271.1
AAT77753.1
ACH89429.1
AAU06852.1
amur_s0994.g1.t1
FAA00746.1
amur_s0008.g13.t1
amur_s0008.g15.t1
amur_s0439.g7.t1
amur_s0439.g9.t1
amur_s0439.g10.t1
amur_s0439.g15.t1
ACH53607.1
0.4

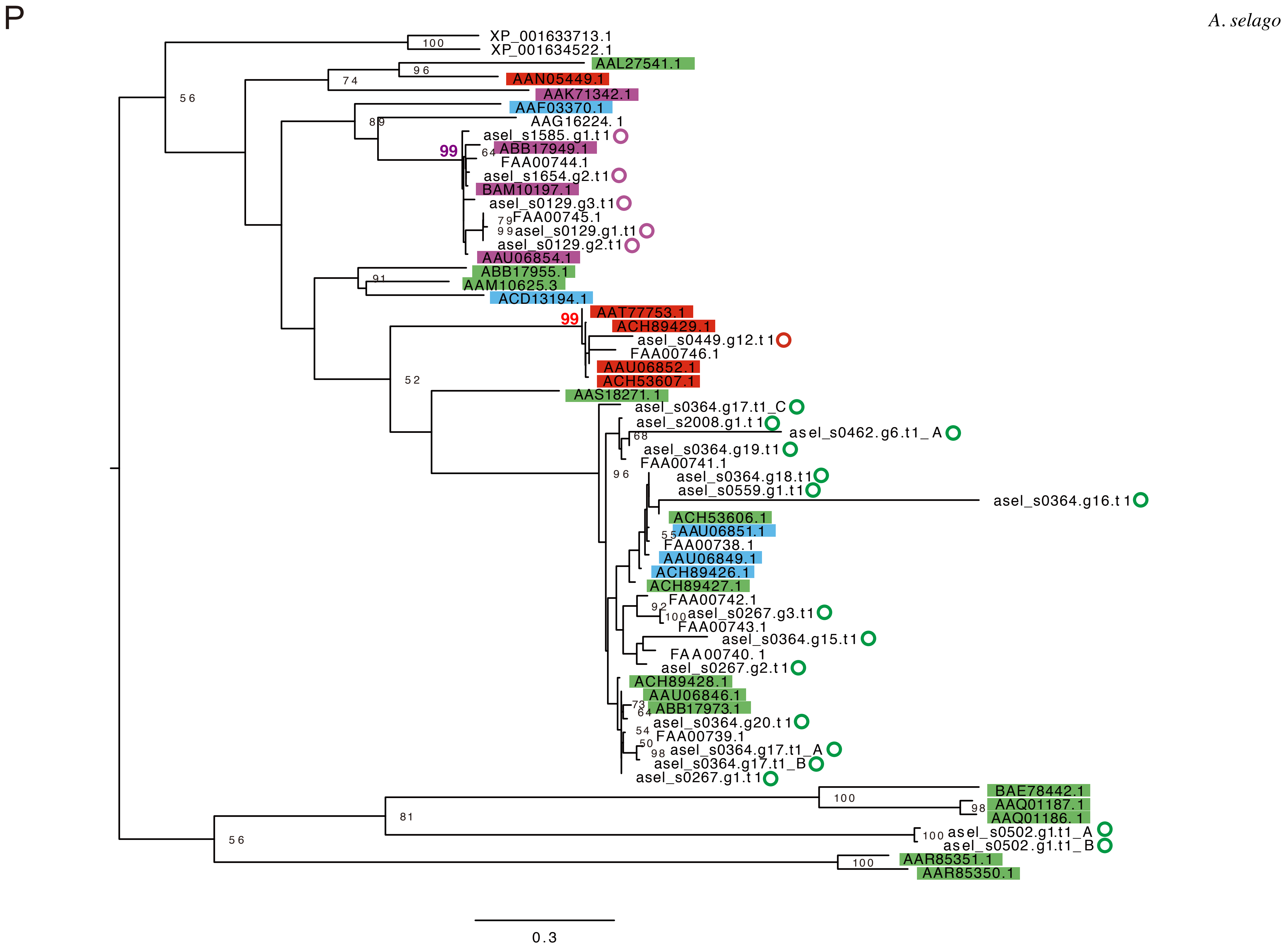
P
A. selago
XP_001633713.1
XP_001634522.1
AAL27541.1
AAN05449.1
AAK71342.1
AAF03370.1
AAG16224.1
asel_s1585.g1.t1
ABB17949.1
FAA00744.1
asel_s1654.g2.t1
BAM10197.1
asel_s0129.g3.t1
FAA00745.1
asel_s0129.g1.t1
asel_s0129.g2.t1
AAU06854.1
ABB17955.1
AAM10625.3
ACD13194.1
AAT77753.1
ACH89429.1
asel_s0449.g12.t1
FAA00746.1
AAU06852.1
ACH53607.1
AAS18271.1
asel_s0364.g17.t1_C
asel_s2008.g1.t1
asel_s0462.g6.t1_A
asel_s0364.g19.t1
FAA00741.1
asel_s0364.g18.t1
asel_s0559.g1.t1
asel_s0364.g16.t1
ACH53606.1
AAU06851.1
FAA00738.1
AAU06849.1
ACH89426.1
ACH89427.1
FAA00742.1
asel_s0267.g3.t1
FAA00743.1
asel_s0364.g15.t1
FAA00740.1
asel_s0267.g2.t1
ACH89428.1
AAU06846.1
ABB17973.1
asel_s0364.g20.t1
FAA00739.1
asel_s0364.g17.t1_A
asel_s0364.g17.t1_B
asel_s0267.g1.t1
BAE78442.1
AAQ01187.1
AAQ01186.1
asel_s0502.g1.t1_A
asel_s0502.g1.t1_B
AAR85351.1
AAR85350.1
0.3

Q

A. cytherea

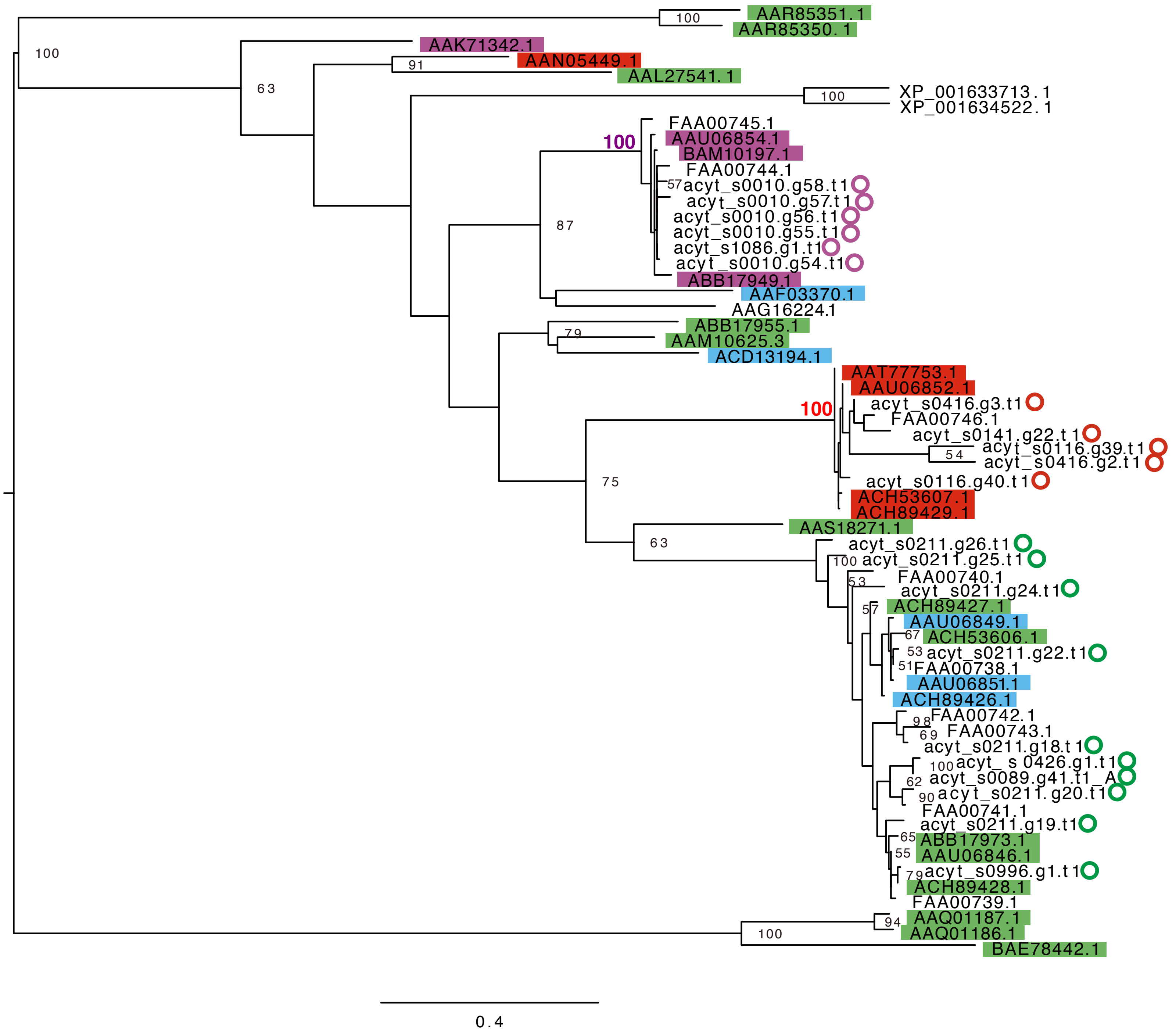
AAR85351.1
AAR85350.1
AAK71342.1
AAN05449.1
AAL27541.1
XP_001633713.1
XP_001634522.1
FAA00745.1
AAU06854.1
BAM10197.1
FAA00744.1
acyt_s0010.g58.t1
acyt_s0010.g57.t1
acyt_s0010.g56.t1
acyt_s0010.g55.t1
acyt_s1086.g1.t1
acyt_s0010.g54.t1
ABB17949.1
AAF03370.1
AAG16224.1
ABB17955.1
AAM10625.3
ACD13194.1
AAT77753.1
AAU06852.1
acyt_s0416.g3.t1
FAA00746.1
acyt_s0141.g22.t1
acyt_s0116.g39.t1
acyt_s0416.g2.t1
acyt_s0116.g40.t1
ACH53607.1
ACH89429.1
AAS18271.1
acyt_s0211.g26.t1
acyt_s0211.g25.t1
FAA00740.1
acyt_s0211.g24.t1
ACH89427.1
AAU06849.1
ACH53606.1
acyt_s0211.g22.t1
FAA00738.1
AAU06851.1
ACH89426.1
FAA00742.1
FAA00743.1
acyt_s0211.g18.t1
acyt_s0426.g1.t1
acyt_s0089.g41.t1_A
acyt_s0211.g20.t1
FAA00741.1
acyt_s0211.g19.t1
ABB17973.1
AAU06846.1
acyt_s0996.g1.t1
ACH89428.1
FAA00739.1
AAQ01187.1
AAQ01186.1
BAE78442.1
0.4

R

A. hyacinthus

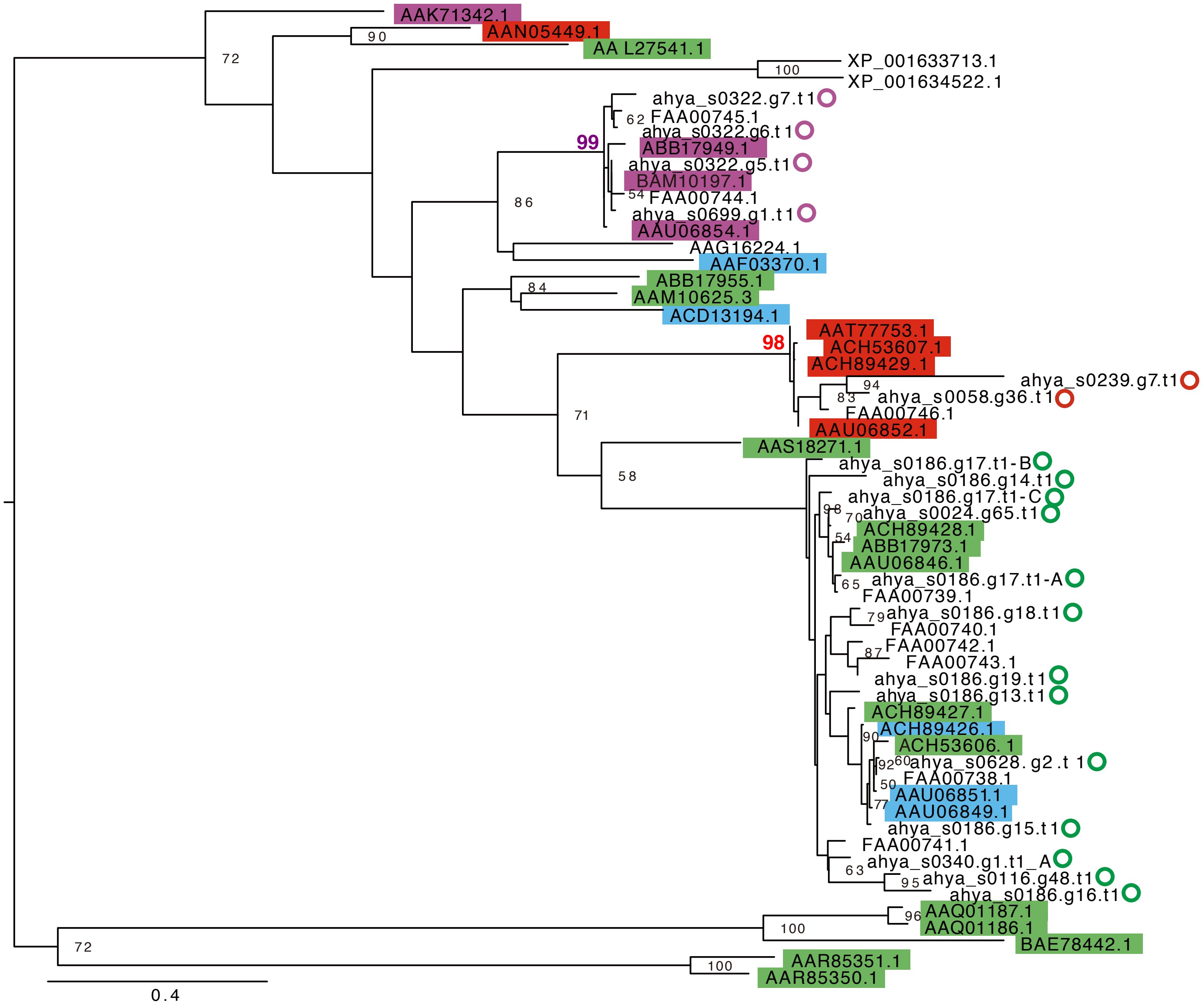

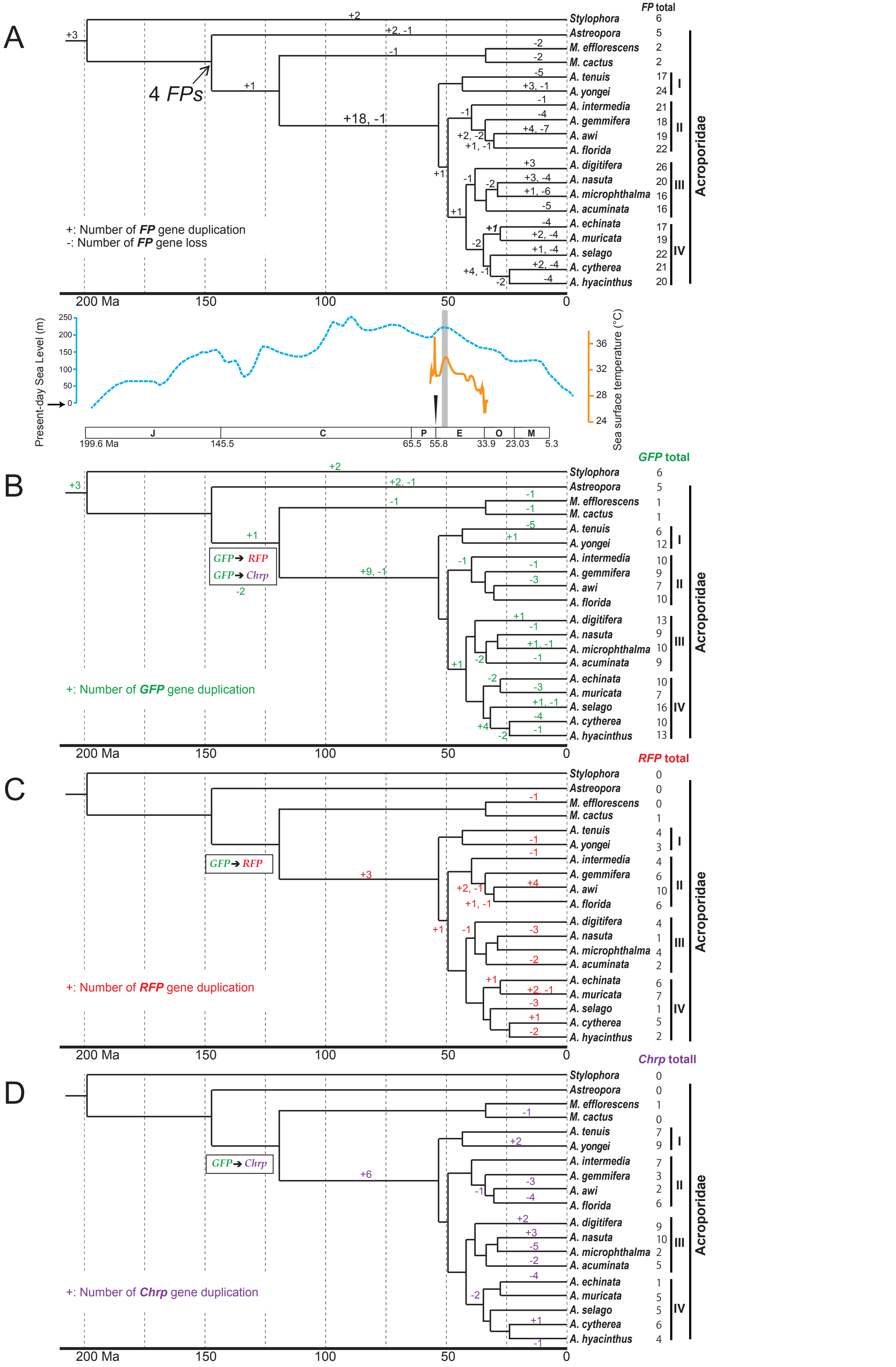

Figure S2. Expansions of fluorescent protein (FP) genes of the genus *Acropora* with estimated divergence times of acroporid corals by preliminary analysis. Numbers of genes duplicated (+) or lost (-) are indicated at branches. (A) FP gene duplications and losses. It is inferred that the common ancestor of Acroporidae had 5 FPs. Species trees with divergence time estimates, changes of sea level, and sea surface temperature are indicated [22]. Expected sea level changes are shown with a blue dotted line [55]. Estimated tropical sea surface temperature of the Eocene is indicated with an orange line [56]. The Paleocene–Eocene Thermal Maximum (PETM) is indicated with an arrowhead, and the Early Eocene Climatic Optimum (EECO) is highlighted in light grey. An approximate geological time scale is shown at the bottom. J: Jurassic Period, C: Cretaceous Period, P: Paleocene, E: Eocene, O: Oligocene, and M: Miocene. The history of FP duplications and losses are classified into those of GFP/CFPs (B), RFPs (C), and Chrps (D). Squares show that putative GFP evolved into RFP or ChrP.

A. GFP/CFP

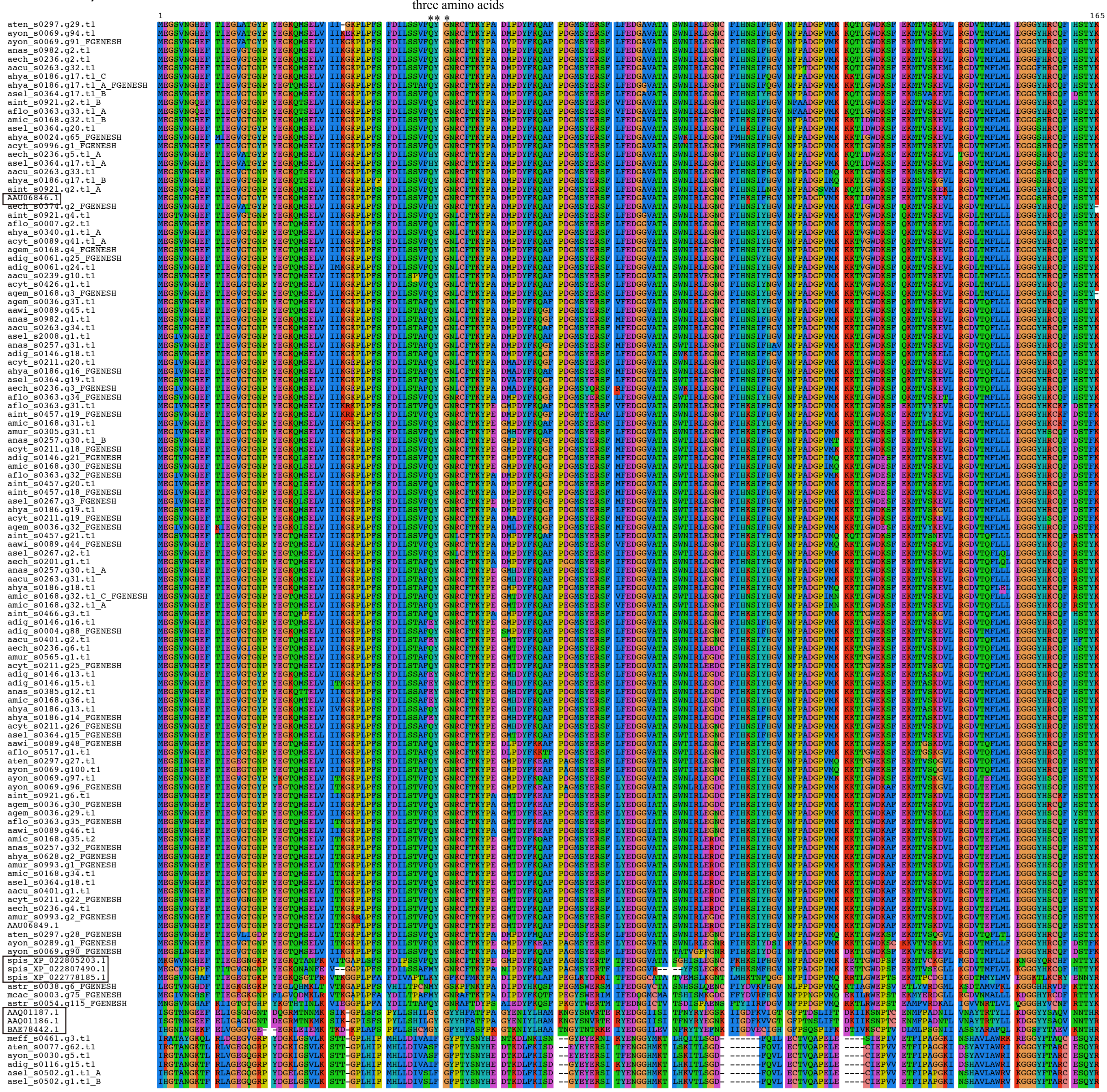

B. RFP

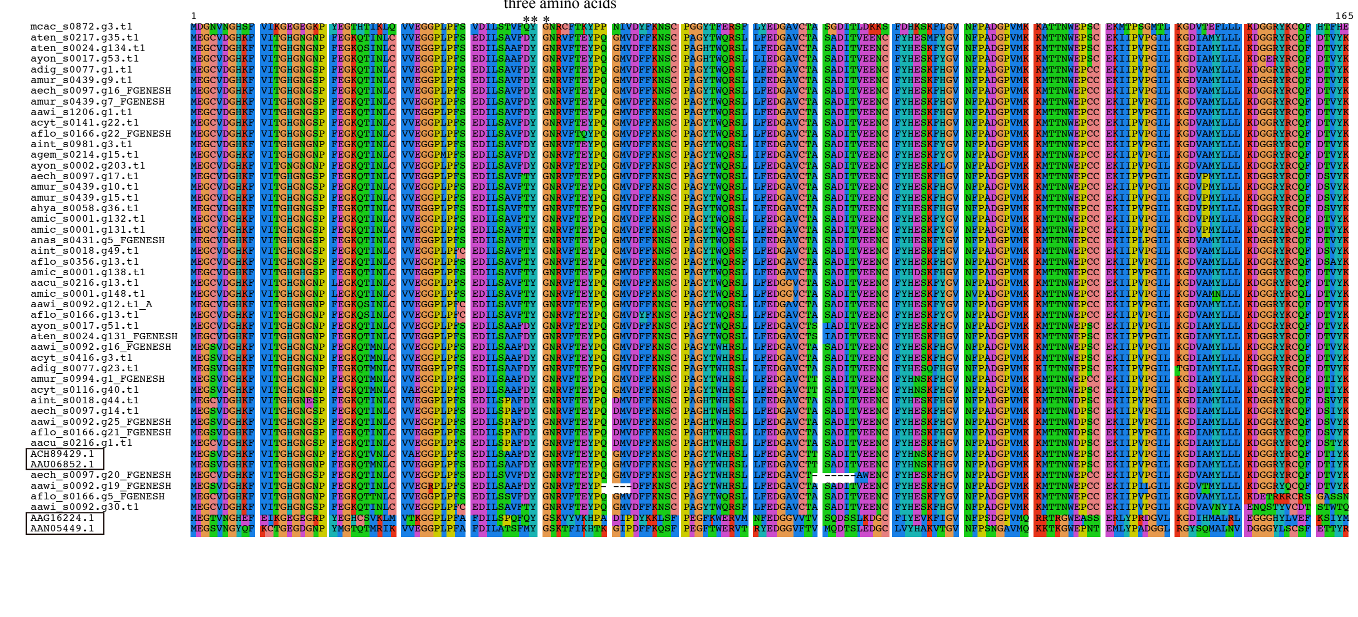

C. Chromoprotein

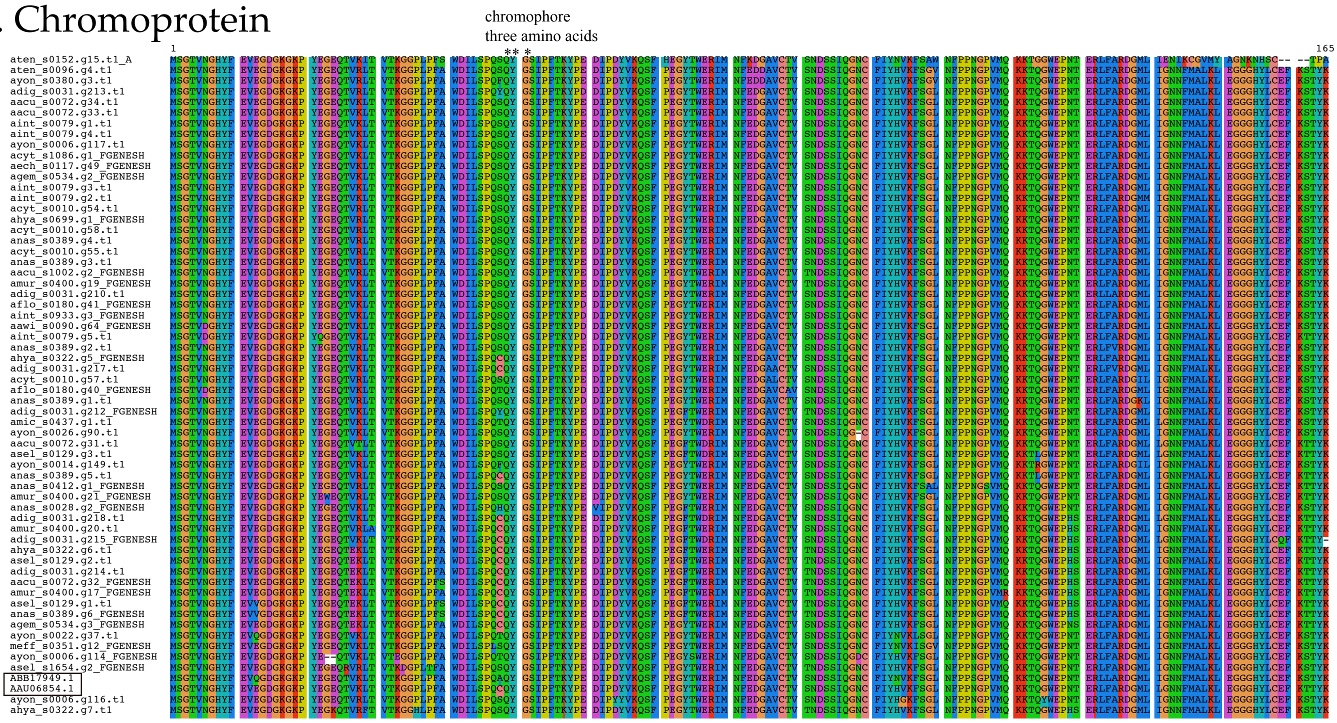

Figure S3. Alignments of predicted FP proteins of 18 acroporid corals. (A) Predicted GFPs/CFPs. (B) Predicted RFPs. (C) Predicted Chrps. Sites of the 3-amino acid chromophores are shown with asterisks. Squares show referred proteins (See also Table 1).

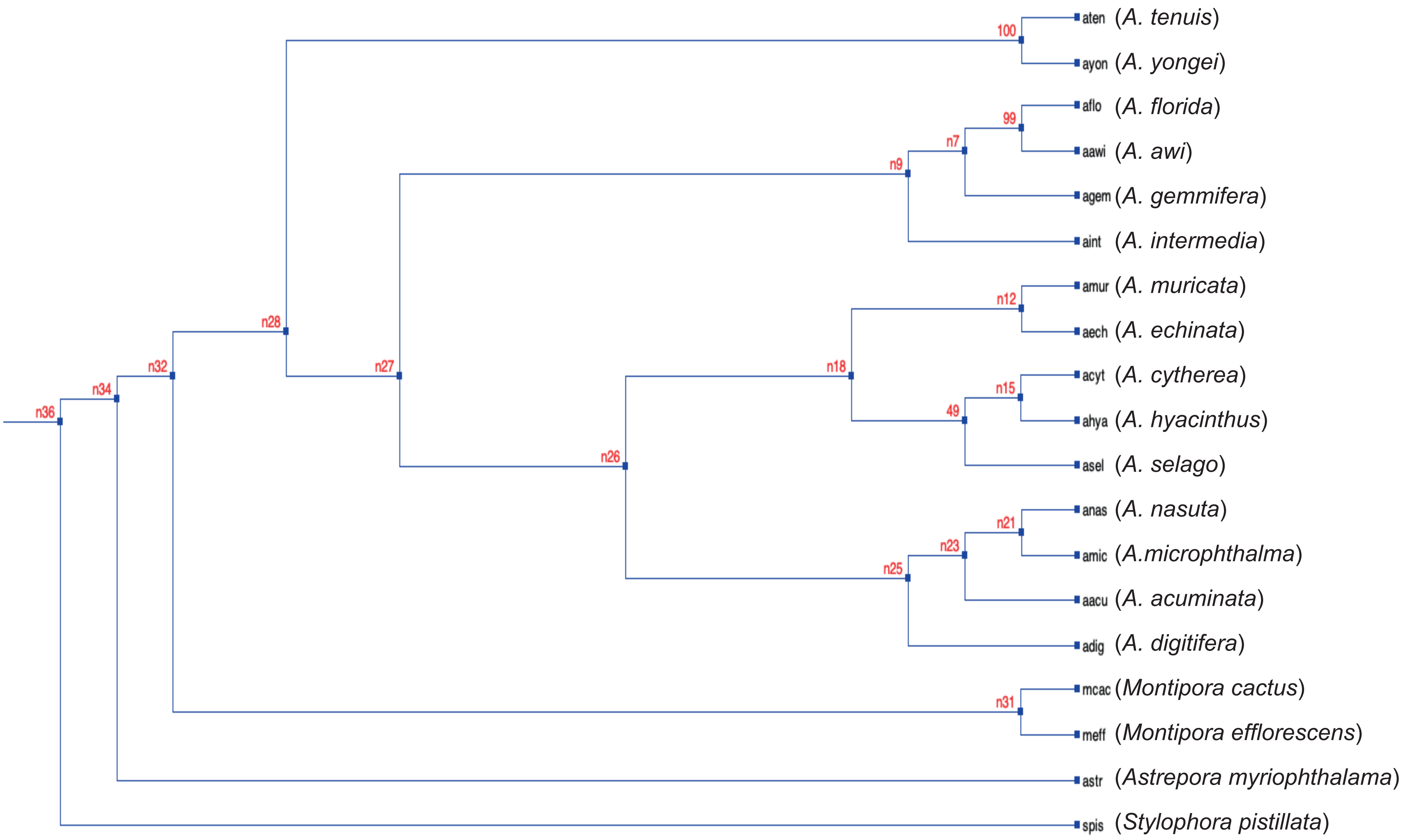

Figure S4. A phylogenetic tree showing node IDs from Notung output. Red letters show node IDs assigned by Notung, as also indicated in Table S3.

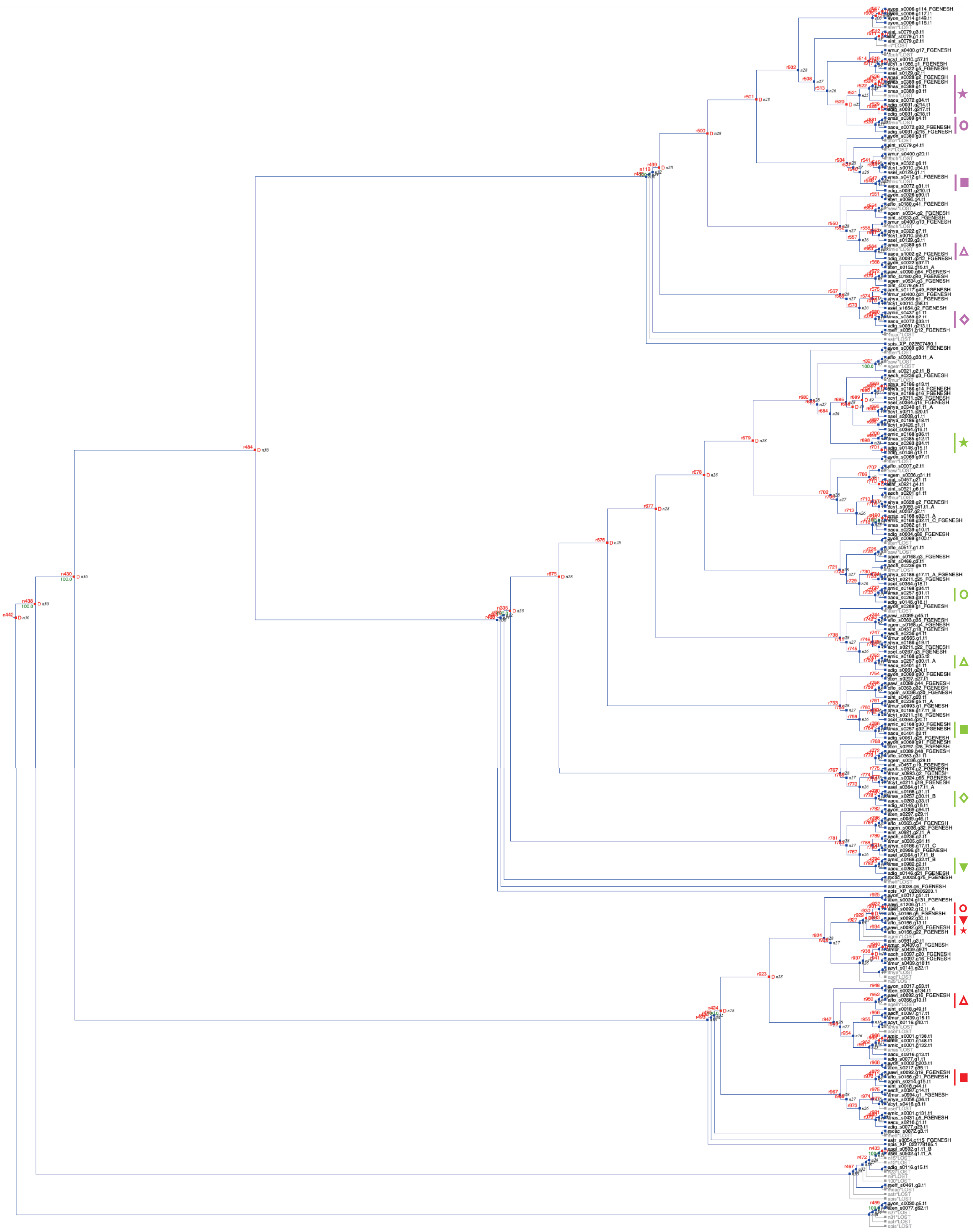

Figure S5. Ortholog prediction by molecular phylogenetic tree of the Acroporidae FPs. Estimated gene duplications are shown by red-letter “D” at internal node. Other red-letters are node IDs that are assigned by Notung software. Estimated gene loss is indicated by gray-letter “node ID and LOST” (see Figure S3 for node ID) at tip. High bootstrap values (100%) were shown in green-letter at internal node. GFP, RFP, and Chrp orthologous groups for *Acropora* species were shown with the marks (asterisk, circle, square, triangle, and lozenge) in green, pink, and purple, respectively. (See also Figure 3).

Table S 1. FP genes for preliminary analysis

Coral species	GFP/CFP		RFP		Chromoprotein	
Astrepora myriophthalama	astr_s0038.g6.t1*	astr_s0111.g1.t1				
	astr_s0038.g10.t1_A	astr_s0038.g11.t1_A				
	astr_s0054.g115.t1					
Montipora efflorescens	meff_s0461.g3.t1				meff_s0351.g12.t1_A	
Montipora cactus	mcac_s0003.g75.t1		mcac_s0872.g3.t1			
A. tenuis	aten_s0010.g7.t1	aten_s0297.g29.t1	aten_s0024.g131.t1	aten_s0025.g63.t1	aten_s0096.g1.t1	aten_s0152.g11.t1
	aten_s0077.g62.t1	aten_s0366.g7.t1	aten_s0024.g134.t1	aten_s0217.g35.t1	aten_s0096.g3.t1	aten_s0152.g15.t1_A
	aten_s0297.g27.t1				aten_s0096.g4.t1	aten_s0182.g24.t1
	aten_s0297.g28.t1				aten_s0152.g9.t1_A	
A. yongei	ayon_s0030.g5.t1	ayon_s0069.g98.t1_A	ayon_s0002.g203.t1		ayon_s0006.g113.t1	ayon_s0022.g37.t1
	ayon_s0069.g90.t1	ayon_s0069.g100.t1	ayon_s0017.g51.t1		ayon_s0006.g114.t1	ayon_s0026.g90.t1
	ayon_s0069.g91.t1	ayon_s0289.g1.t1	ayon_s0017.g53.t1		ayon_s0006.g115.t1	ayon_s0380.g3.t1
	ayon_s0069.g93.t1	ayon_s0289.g2.t1			ayon_s0006.g116.t1	
	ayon_s0069.g94.t1	ayon_s0069.g96.t1			ayon_s0006.g117.t1	
	ayon_s0069.g95.t1	ayon_s0069.g97.t1			ayon_s0014.g149.t1	
A. intermedia	aint_s0457.g18.t1	aint_s0921.g1.t1	aint_s0018.g44.t1		aint_s0079.g1.t1	aint_s0933.g3.t1
	aint_s0457.g19.t1	aint_s0921.g2.t1_A	aint_s0018.g49.t1		aint_s0079.g2.t1	aint_s5099.g1.t1
	aint_s0457.g20.t1	aint_s0921.g2.t1_B	aint_s0981.g3.t1		aint_s0079.g3.t1	
	aint_s0457.g21.t	aint_s0921.g4.t1	aint_s2084.g1.t1		aint_s0079.g4.t1	
	aint_s0466.g3.t1	aint_s0921.g6.t1			aint_s0079.g5.t1	
A. gemmifera	agem_s0036.g28.t1	agem_s0232.g2.t1	agem_s0214.g4.t1	agem_s0676.g1.t1	agem_s0534.g2.t1	
	agem_s0036.g31.t1	agem_s0927.g1.t1	agem_s0214.g5.t1		agem_s0534.g3.t1	
	agem_s0036.g32.t1	agem_s0036.g29.t1	agem_s0214.g12.t1		agem_s0837.g1.t1	
	agem_s0168.g3.t1	agem_s0036.g30.t1	agem_s0214.g15.t1			
	agem_s0168.g4.t1		agem_s0214.g24.t1			
A. awi	aawi_s0089.g43.t1	aawi_s0089.g49.t1	aawi_s0092.g11.t1	aawi_s0092.g19.t1	aawi_s0090.g64.t1	
	aawi_s0089.g44.t1	aawi_s0089.g46.t1	aawi_s0092.g12.t1_A	aawi_s0092.g25.t1	aawi_s0090.g65.t1	
	aawi_s0089.g45.t1		aawi_s0092.g16.t1	aawi_s0092.g26.t1		
	aawi_s0089.g47.t1		aawi_s0092.g17.t1	aawi_s0092.g30.t1		
	aawi_s0089.g48.t1		aawi_s0092.g18.t1_A	aawi_s1206.g1.t1		
	aflo_s0007.g2.t1	aflo_s0363.g34.t1	aflo_s0166.g5.t1	aflo_s0356.g14.t1	aflo_s0012.g118.t1	aflo_s0180.g42.t1
A. florida	aflo_s0363.g31.t1	aflo_s0363.g35.t1	aflo_s0166.g13.t1		aflo_s0012.g119.t1	
	aflo_s0363.g32.t1	aflo_s0363.g36.t1	aflo_s0166.g21.t1		aflo_s0180.g39.t1	
	aflo_s0363.g33.t1_A	aflo_s0517.g1.t1	aflo_s0166.g22.t1		aflo_s0180.g40.t1	
	aflo_s0363.g33.t1_A	aflo_s1975.g1.t1	aflo_s0356.g13.t1		aflo_s0180.g41.t1	
A. digitifera	adig_s0004.g88.t1	adig_s0146.g16.t1	adig_s0077.g1.t1		adig_s0031.g210.t1	adig_s0031.g216.t1
	adig_s0061.g24.t1	adig_s0146.g17.t1	adig_s0077.g2.t1		adig_s0031.g211.t1	adig_s0031.g217.t1
	adig_s0061.g25.t1	adig_s0146.g18.t1	adig_s0077.g3.t1		adig_s0031.g212.t1	adig_s0031.g218.t1
	adig_s0116.g15.t1	adig_s0146.g19.t1	adig_s0077.g23.t1		adig_s0031.g213.t1	
	adig_s0146.g13.t1	adig_s0146.g20.t1			adig_s0031.g214.t1	
	adig_s0146.g14.t1	adig_s0146.g21.t1			adig_s0031.g215.t1	
	adig_s0146.g15.t1					
A. nasuta	anas_s0191.g1.t1	anas_s0385.g12.t1	anas_s0431.g5.t1		anas_s0028.g1.t1	anas_s0389.g3.t1
	anas_s0191.g2.t1	anas_s0982.g1.t1			anas_s0028.g2.t1	anas_s0389.g4.t1
	anas_s0257.g30.t1_A	anas_s0982.g2.t1			anas_s0028.g3.t1	anas_s0389.g5.t1
	anas_s0257.g30.t1_B	anas_s0257.g32.t1			anas_s0389.g1.t1	anas_s0389.g6.t1
	anas_s0257.g31.t1	anas_s0982.g2.t1*			anas_s0389.g2.t1	anas_s0412.g1.t1
A. microphthalma	amic_s0168.g30.t1	amic_s0168.g33.t1	amic_s0001.g131.t1		amic_s0437.g1.t1	
	amic_s0168.g31.t1	amic_s0168.g35.t1	amic_s0001.g132.t1		amic_s0437.g2.t1	
	amic_s0168.g32.t1_A	amic_s0168.g36.t1	amic_s0001.g138.t1			
	amic_s0168.g32.t1_B	amic_s3224.g1.t1	amic_s0001.g148.t1			
	amic_s0168.g32.t1_C	amic_s0168.g34.t1				
A. acuminata	aacu_s0239.g9.t1	aacu_s0263.g34.t1	aacu_s0216.g1.t1		aacu_s0072.g31.t1	aacu_s1002.g2.t1
	aacu_s0239.g10.t1	aacu_s0401.g2.t1	aacu_s0216.g13.t1		aacu_s0072.g32.t1	
	aacu_s0263.g31.t1	aacu_s0401.g3.t1_A			aacu_s0072.g33.t1	
	aacu_s0263.g32.t1	aacu_s0401.g1.t1			aacu_s0072.g34.t1	
	aacu_s0263.g33.t1					
A. echinata	aech_s0054.g127.t1	aech_s0236.g3.t1	aech_s0086.g75.t1	aech_s0359.g4.t1_A	aech_s0117.g49.t1	
	aech_s0201.g1.t1	aech_s0236.g5.t1_A	aech_s0097.g14.t1			
	aech_s0201.g2.t1	aech_s0236.g6.t1	aech_s0097.g16.t1			
	aech_s0236.g1.t1	aech_s0374.g2.t1	aech_s0097.g17.t1			
	aech_s0236.g2.t1	aech_s0236.g4.t1	aech_s0097.g20.t1			
A. muricata	amur_s0305.g31.t1	amur_s0993.g3.t1	amur_s0008.g13.t1	amur_s0439.g9.t1	amur_s0400.g17.t1	amur_s0400.g21.t1
	amur_s0305.g32.t1	amur_s0993.g1.t1	amur_s0008.g15.t1	amur_s0439.g10.t1	amur_s0400.g18.t1	
	amur_s0565.g1.t1	amur_s0993.g2.t1	amur_s0994.g1.t1	amur_s0439.g15.t1	amur_s0400.g19.t1	
	amur_s0815.g2.t1		amur_s0439.g7.t1		amur_s0400.g20.t1	
A. selago	asel_s0267.g1.t1	asel_s0364.g19.t1	asel_s0449.g12.t1		asel_s0129.g1.t1	
	asel_s0267.g2.t1	asel_s0364.g20.t1			asel_s0129.g2.t1	
	asel_s0267.g3.t1	asel_s0462.g6.t1_A			asel_s0129.g3.t1	
	asel_s0364.g15.t1	asel_s0502.g1.t1_A			asel_s1585.g1.t1	
	asel_s0364.g16.t1	asel_s0502.g1.t1_B			asel_s1654.g2.t1	
	asel_s0364.g17.t1_A	asel_s2008.g1.t1				
	asel_s0364.g17.t1_A	asel_s0364.g18.t1				
	asel_s0364.g17.t1_B	asel_s0559.g1.t1				
A. cytherea	acyt_s0089.g41.t1_A	acyt_s0211.g24.t1	acyt_s0116.g39.t1		acyt_s0010.g54.t1	acyt_s1086.g1.t1
	acyt_s0211.g18.t1	acyt_s0211.g25.t1	acyt_s0116.g40.t1		acyt_s0010.g55.t1	
	acyt_s0211.g19.t1	acyt_s0211.g26.t1	acyt_s0141.g22.t1		acyt_s0010.g56.t1	
	acyt_s0211.g20.t1	acyt_s0426.g1.t1	acyt_s0416.g2.t1		acyt_s0010.g57.t1	
	acyt_s0211.g22.t1	acyt_s0996.g1.t1	acyt_s0416.g3.t1		acyt_s0010.g58.t1	
A. hyacinthus	ahya_s0024.g65.t1	ahya_s0186.g17.t1_C	ahya_s0058.g36.t1		ahya_s0322.g5.t1	
	ahya_s0116.g48.t1	ahya_s0186.g18.t1	ahya_s0239.g7.t1		ahya_s0322.g6.t1	
	ahya_s0186.g13.t1	ahya_s0186.g19.t1			ahya_s0322.g7.t1	
	ahya_s0186.g14.t1	ahya_s0340.g1.t1_A			ahya_s0699.g1.t1	
	ahya_s0186.g16.t1	ahya_s0186.g15.t1				
	ahya_s0186.g17.t1_A	ahya_s0628.g2.t1				
	ahya_s0186.g17.t1_B					

*The number following "s" in gene model ID indicates a scaffold number that the gene is located on. For example, "astr_s0038.g6.t1" is mapped on scaffold 38.

Table S 2. Analyzed FP genes

Coral species	GFP/CFP		RFP		Chromoprotein	
Astrepora myriophthalama	astr_s0038.g6_FGENESH	astr_s0054.g115_FGENESH				
Montipora efflorescens	meff_s0461.g3.t1				meff_s0351.g12_FGENESH	
Montipora cactus	mcac_s0003.g75_FGENESH		mcac_s0872.g3.t1			
A. tenuis	aten_s0077.g62.t1	aten_s0297.g28_FGENESH	aten_s0024.g131_FGENESH	aten_s0217.g35.t1	aten_s0096.g4.t1	
	aten_s0297.g27.t1	aten_s0297.g29.t1	aten_s0024.g134.t1		aten_s0152.g15.t1_A	
A. yongei	ayon_s0030.g5.t1	ayon_s0069.g96_FGENESH	ayon_s0002.g203.t1		ayon_s0006.g114_FGENESH	ayon_s0022.g37.t1
	ayon_s0069.g90_FGENESH	ayon_s0069.g97.t1	ayon_s0017.g51.t1		ayon_s0006.g116.t1	ayon_s0026.g90.t1
	ayon_s0069.g91_FGENESH	ayon_s0069.g100.t1	ayon_s0017.g53.t1		ayon_s0006.g117.t1	ayon_s0380.g3.t1
	ayon_s0069.g94.t1	ayon_s0289.g1_FGENESH			ayon_s0014.g149.t1	
A. intermedia	aint_s0457.g18_FGENESH	aint_s0921.g2.t1_A	aint_s0018.g44.t1		aint_s0079.g1.t1	aint_s0933.g3_FGENESH
	aint_s0457.g19_FGENESH	aint_s0921.g2.t1_B	aint_s0018.g49.t1		aint_s0079.g2.t1	
	aint_s0457.g20.t1	aint_s0921.g4.t1	aint_s0981.g3.t1		aint_s0079.g3.t1	
	aint_s0457.g21.t	aint_s0921.g6.t1			aint_s0079.g4.t1	
	aint_s0466.g3.t1				aint_s0079.g5.t1	
A. gemmifera	agem_s0036.g29.t1	agem_s0036.g31.t1	agem_s0214.g15.t1		agem_s0534.g2_FGENESH	
	agem_s0036.g30_FGENESH	agem_s0168.g3_FGENESH			agem_s0534.g3_FGENESH	
	agem_s0036.g32_FGENESH	agem_s0168.g4_FGENESH				
A. awi	aawi_s0089.g44_FGENESH	aawi_s0089.g48_FGENESH	aawi_s0092.g12.t1_A	aawi_s0092.g25_FGENESH	aawi_s0090.g64_FGENESH	
	aawi_s0089.g45.t1		aawi_s0092.g16_FGENESH	aawi_s0092.g30.t1		
	aawi_s0089.g46.t1		aawi_s0092.g19_FGENESH	aawi_s1206.g1.t1		
A. florida	aflo_s0007.g2.t1	aflo_s0363.g34_FGENESH	aflo_s0166.g5_FGENESH	aflo_s0356.g13.t1	aflo_s0180.g40_FGENESH	
	aflo_s0363.g31.t1	aflo_s0363.g35_FGENESH	aflo_s0166.g13.t1		aflo_s0180.g41_FGENESH	
	aflo_s0363.g32_FGENESH	aflo_s0517.g1.t1	aflo_s0166.g21_FGENESH			
	aflo_s0363.g33.t1_A		anas_s0431.g5_FGENESH			
A. digitifera	adig_s0004.g88_FGENESH	adig_s0146.g15.t1	adig_s0077.g1.t1		adig_s0031.g210.t1	adig_s0031.g217.t1
	adig_s0061.g24.t1	adig_s0146.g16.t1	adig_s0077.g23.t1		adig_s0031.g212_FGENESH	adig_s0031.g218.t1
	adig_s0061.g25_FGENESH	adig_s0146.g18.t1			adig_s0031.g213.t1	
	adig_s0116.g15.t1	adig_s0146.g21_FGENESH			adig_s0031.g214.t1	
	adig_s0146.g13.t1				adig_s0031.g215_FGENESH	
A. nasuta	anas_s0257.g30.t1_A	aacu_s0263.g32.t1	anas_s0431.g5_FGENESH		anas_s0028.g2_FGENESH	anas_s0389.g4.t1
	anas_s0257.g30.t1_B	anas_s0385.g12.t1			anas_s0389.g1.t1	anas_s0389.g5.t1
	anas_s0257.g31.t1	anas_s0982.g1.t1			anas_s0389.g2.t1	anas_s0389.g6_FGENESH
	anas_s0257.g32_FGENESH				anas_s0389.g3.t1	anas_s0412.g1_FGENESH
A. microphthalma	amic_s0168.g30_FGENESH	amic_s0168.g32.t1_C_FGENI	amic_s0001.g131.t1		amic_s0437.g1.t1	
	amic_s0168.g31.t1	amic_s0168.g34.t1	amic_s0001.g132.t1			
	amic_s0168.g32.t1_A	amic_s0168.g35.t2	amic_s0001.g138.t1			
	amic_s0168.g32.t1_B	amic_s0168.g36.t1	amic_s0001.g148.t1			
A. acuminata	aacu_s0239.g10.t1	aacu_s0263.g34.t1	aacu_s0216.g1.t1		aacu_s0072.g31.t1	aacu_s1002.g2_FGENESH
	aacu_s0263.g31.t1	aacu_s0401.g1.t1	aacu_s0216.g13.t1		aacu_s0072.g32_FGENESH	
	aacu_s0263.g32.t1	aacu_s0401.g2.t1			aacu_s0072.g33.t1	
	aacu_s0263.g33.t1				aacu_s0072.g34.t1	
A. echinata	aech_s0201.g1.t1	aech_s0236.g5.t1_A	aech_s0097.g14.t1		aech_s0117.g49_FGENESH	
	aech_s0236.g2.t1	aech_s0236.g6.t1	aech_s0097.g17.t1			
	aech_s0236.g3_FGENESH	aech_s0374.g2_FGENESH	aech_s0097.g16_FGENESH			
	aech_s0236.g4.t1		aech_s0097.g20_FGENESH			
A. muricata	amur_s0305.g31.t1	amur_s0993.g2_FGENESH	amur_s0439.g7_FGENESH	amur_s0439.g15.t1	amur_s0400.g17_FGENESH	amur_s0400.g21_FGENESH
	amur_s0565.g1.t1		amur_s0439.g9.t1	amur_s0994.g1_FGENESH	amur_s0400.g19_FGENESH	
	amur_s0993.g1_FGENESH		amur_s0439.g10.t1		amur_s0400.g20.t1	
A. selago	asel_s0267.g2.t1	asel_s0364.g19.t1			asel_s0129.g1.t1	
	asel_s0267.g3_FGENESH	asel_s0364.g20.t1			asel_s0129.g2.t1	
	asel_s0364.g15_FGENESH	asel_s0502.g1.t1_A			asel_s0129.g3.t1	
	asel_s0364.g17.t1_A	asel_s0502.g1.t1_B			asel_s1654.g2_FGENESH	
	asel_s0364.g17.t1_B	asel_s2008.g1.t1				
	asel_s0364.g18.t1					
A. cytherea	acyt_s0089.g41.t1_A	acyt_s0211.g25_FGENESH	acyt_s0116.g40.t1		acyt_s0010.g54.t1	
	acyt_s0211.g18_FGENESH	acyt_s0211.g26_FGENESH	acyt_s0141.g22.t1		acyt_s0010.g55.t1	
	acyt_s0211.g19_FGENESH	acyt_s0426.g1.t1	acyt_s0416.g3.t1		acyt_s0010.g57.t1	
	acyt_s0211.g20.t1	acyt_s0996.g1_FGENESH			acyt_s0010.g58.t1	
	acyt_s0211.g22_FGENESH				acyt_s1086.g1_FGENESH	
A. hyacinthus	ahya_s0024.g65_FGENESH	ahya_s0186.g17.t1_C	ahya_s0058.g36.t1		ahya_s0322.g5_FGENESH	
	ahya_s0186.g13.t1	ahya_s0186.g18.t1			ahya_s0322.g6.t1	
	ahya_s0186.g14_FGENESH	ahya_s0186.g19.t1			ahya_s0322.g7.t1	
	ahya_s0186.g16_FGENESH	ahya_s0340.g1.t1_A			ahya_s0699.g1_FGENESH	
	ahya_s0186.g17.t1_A_FGENI	ahya_s0628.g2_FGENESH				
	ahya_s0186.g17.t1_B					

Table S3. Analyzed FP duplications and losses

All			GFP/ CFP			RFP			Chromoprotein		
Node IDs *	Duplications	Losses	Node IDs	Duplications	Losses	Node IDs	Duplications	Losses	Node IDs	Duplications	Losses
n9	0	1	spis	0	2	adig	0	0	99	0	0
n18	0	0	n18	0	0	n7	0	0	n18	0	0
ayon	3	0	asel	1	0	anas	0	1	n21	0	0
aacu	0	0	100	0	1	n28	2	0	aawi	0	1
amur	1	3	n21	0	0	aech	0	0	asel	0	0
n28	11	0	acyt	0	0	meff	0	1	100	0	0
99	2	0	ayon	0	0	asel	0	3	n26	0	0
100	0	1	n32	0	0	amur	1	0	n23	0	0
n32	0	0	anas	0	0	aten	0	0	adig	2	0
n12	1	1	aint	2	0	aint	0	0	49	0	0
n23	0	1	n15	0	1	n23	0	0	n12	0	0
ahya	2	2	n28	6	0	49	0	0	aacu	0	0
n21	0	0	n12	0	1	n27	0	0	n9	0	0
aten	0	6	99	0	0	n9	0	0	acyt	1	0
n34	0	0	aawi	0	3	agem	0	2	ahya	0	0
n26	0	0	adig	1	0	amic	2	0	n32	0	0
aawi	1	4	n23	0	1	n18	0	0	aten	0	2
n27	0	1	aten	0	4	n32	0	0	n25	1	0
aint	4	0	amur	0	3	n31	0	0	n27	0	0
n36	4	0	n7	0	0	n26	0	0	aint	2	0
spis	0	2	aflo	0	0	n25	0	1	ayon	3	0
n25	1	1	n27	0	1	n12	1	0	agem	0	0
anas	3	1	aacu	0	0	ayon	0	0	aech	0	3
mcac	0	2	astr	0	3	ahya	0	2	n7	0	2
49	2	0	agem	0	1	aawi	1	0	n28	3	0
asel	1	3	meff	0	1	n21	0	0	amic	0	4
aech	0	3	49	2	0	n15	0	0	aflo	0	0
aflo	0	0	n26	0	0	99	2	0	meff	0	0
amic	3	4	n9	0	1	aacu	0	0	n31	0	0
meff	0	2	mcac	0	1	aflo	0	0	amur	0	0
adig	3	0	n36	4	0	mcac	0	0	n15	0	0
astr	0	3	aech	0	0	acyt	0	0	mcac	0	1
n15	0	1	n31	0	1	100	0	0	anas	3	0
agem	0	3	amic	1	0	Duplications: 9			Duplications: 15		
n7	0	2	ahya	2	0	Losses: 10			Losses: 13		
n31	0	1	n25	0	0						
acyt	1	0	n34	0	0						
Duplications: 43			Duplications: 18								
Losses: 48			Losses: 27								

*Node IDs and OTU (operational taxonomic unit) are shown in Figure S3.
#The numbers are shown in Figure 2A.
